# Variation in Ants’ Chemical Recognition Signals across Vineyard Agroecosystems

**DOI:** 10.3390/ijms251910407

**Published:** 2024-09-27

**Authors:** Arthur Hais, Luca Pietro Casacci, Patrizia d’Ettorre, David Badía-Villas, Chloé Leroy, Francesca Barbero

**Affiliations:** 1 Department of Life Sciences and Systems Biology, University of Turin, Via Accademia Albertina 13, 10123 Turin, Italy; arthur.hais@unito.it (A.H.); luca.casacci@unito.it (L.P.C.); 2 Laboratory of Experimental and Comparative Ethology (LEEC), UR4443, University Sorbonne Paris Nord, 93430 Villetaneuse, France; d-ettorre@univ-paris13.fr (P.d.); leroy@univ-paris13.fr (C.L.); 3 Department of Agriculture and Natural Sciences, Escuela Politécnica Superior, 22071 Huesca, Spain; badia@unizar.es

**Keywords:** cuticular hydrocarbons, *Lasius paralienus*, vineyards, soil respiration, organic agriculture

## Abstract

Ant evolutionary success depends mainly on the coordination of colony members, who recognize nestmates based on the cuticular hydrocarbon (CHC) profile of their epicuticle. While several studies have examined variations in this crucial factor for colony identity, few have investigated the anthropic impact on CHC profiles, and none have focused on *Lasius paralienus*. Here, we surveyed the changes in *L. paralienus* CHC assemblages across agroecosystems and assessed whether different vineyard management influences these profiles. Soil sampling within ant nests and in close surroundings was performed to measure microhabitat variations. Our results show that the cuticular chemical composition of *Lasius paralienus* is mainly affected by the differences between areas, with an existing but unclear anthropic influence on them. Normalized soil respiration partially explains these interarea variations. Irrespective of the conventional or organic management, human activities in agroecosystems mostly impacted *L. paralienus* linear alkanes, a specific class of CHCs known to play a major role against dehydration, but also affected the abundance of compounds that can be pivotal for maintaining the colony identity. Our findings suggest that vineyard practices primarily affect features of the ant cuticle, potentially enhancing microclimate adaptations. Still, the potential effects as disruptive factors need further investigation through the implementation of behavioral bioassays.

## 1. Introduction

Ants are among Earth’s richest, most diverse, and abundant animal groups. Their evolutionary success is primarily due to their social organization maintained through a complex communication system, which provides the colony with the plasticity to respond to environmental changes [1,2] promptly. The ability to coordinate hundreds of individuals relies on multimodal [3], chemical [4], tactile [1], visual [5], and vibro-acoustical [3] signals. The nestmate recognition system is the pivotal mechanism underpinning the ants’ eusocial structure and ensuring that altruistic behavior is addressed toward related receivers [6,7]. Group or colony cues permit individuals to distinguish between kin and non-kin [8]. Chemical communication based on cuticular hydrocarbons (CHCs) is the most prominent way ants discriminate between colony members and “strangers” [7,9,10,11,12]. CHCs are long-chain hydrocarbons present on most insect cuticles characterized by low volatility at ambient temperature. These integuments act as a physical barrier; therefore, CHCs are primarily responsible for preventing desiccation but are also crucial sources of chemical signaling [11,13]. They can be separated into different structural classes with a decreasing frequency of occurrence in ant species: *n-*alkanes, monomethylalkanes, dimethylalkanes, alkenes, dienes, trimethylalkanes, methylalkenes, methylalkadienes, trienes, and tetramethylalkanes [14].

Although the exact function of each compound class and how the recognition system is achieved are far from being fully disentangled, some authors reported that blends of at least two CHC classes are fundamental to eliciting a recognition response in ants [15]. Each individual possesses a CHC profile described by the proportion and abundance of several compounds on its epicuticle. By smelling other “items” at short distances with the antennae, an ant inspects their chemical profiles and compares them to its own [16], which therefore functions as a template [9,17,18]. Nestmates are then ignored, whereas intruders often prompt antagonistic behaviors [19,20].

CHC signatures can vary in the number of compounds, their respective proportions, chain lengths, and chemical compositions across species, population, or colony levels [21]. As each species usually possesses the same CHCs, the intra-specific nestmate recognition is mainly based on quantitative variations in the CHCs [9]. Still, these variations are mitigated inside each colony by trophallaxis and allogrooming behaviors, which create a homogenized CHC colony odor [9,22,23].

Because CHC profiles are at least partially genetically determined [24], qualitative variations can be due to gene expression regulation rather than gene loss [25]. In addition, the exogenous uptake of specific amino acids through nutrition affects the biosynthesis of certain CHCs. Thus, the CHC profile variations can be caused by genetic as well as biotic and abiotic environmental factors such as temperature, humidity, nest material, diet, or microbiome [21,26]. In addition, cuticular compounds can also entrap lipophilic substances from the environment [27], acting as indicators of pollutants.

Habitat modifications, such as agricultural practices, heavily impact the inhabiting insect communities, notably through chemicals or land-cover changes [28,29]. Among arthropods, ants are extremely sensitive to these habitat modifications [30,31,32], especially the terrestrial species that rely on soil as the primary material for nest construction. However, the properties of this nest material influence CHC profiles in an undetermined way [21]. Even if the impact of environmental factors, such as temperature or humidity, on CHC profiles has been previously investigated on ants [21,33,34,35], only one study performed on social wasps explored how the anthropic modifications influence the chemical epicuticular signature revealing quali-quantitative changes [36].

Changes in the nestmate recognition system caused by impactful agricultural methods can reduce the ecosystem services provided by insects, including ants, in cultivated areas. Therefore, our study aims to assess if and how organic and conventional agricultural practices affect the CHC profiles of a soil-nesting ant species, *Lasius paralienus,* which was widespread in all studied areas. According to Seifert [37], *L. paralienus* occurs mostly in the xerothermophilous meadows of central Europe and the Mediterranean region. Colonies are probably monogynous, and nests are built in the soil, under rocks, or in the root turf, lacking conspicuous solaria.

We analyzed the CHC variation among *L. paralienus* ant colonies collected in three areas from vineyards managed with different agronomic practices and from uncultivated fields (Figure S1).

Colonies of *L. paralienus* from the same area should have more similar colony odors than colonies from different areas because they share the same environmental conditions, and even slight geographic distances can affect CHCs quantitatively [38,39]. However, we hypothesize that the magnitude of the anthropic impact, described as different management practices, overcomes the geographical effect. Therefore, we expect higher dissimilarities in the CHC profiles of ant colonies from the same area sampled in vineyards distinctly managed than between ant colonies gathered in vineyards with the same management regime from different geographic areas.

In addition, because organic agriculture employs fewer agrochemicals than conventional agriculture, thus preserving a higher species richness and likely impacting less on biological colony traits [40,41,42,43], we foresee a closer overlap between the CHC profile of ants collected in the organic vineyards and natural areas than with those inhabiting conventional fields.

## 2. Results

### 2.1. Soil Sampling Results

#### 2.1.1. Soil’s Main Chemical and Physical Properties

**Figure 1:**
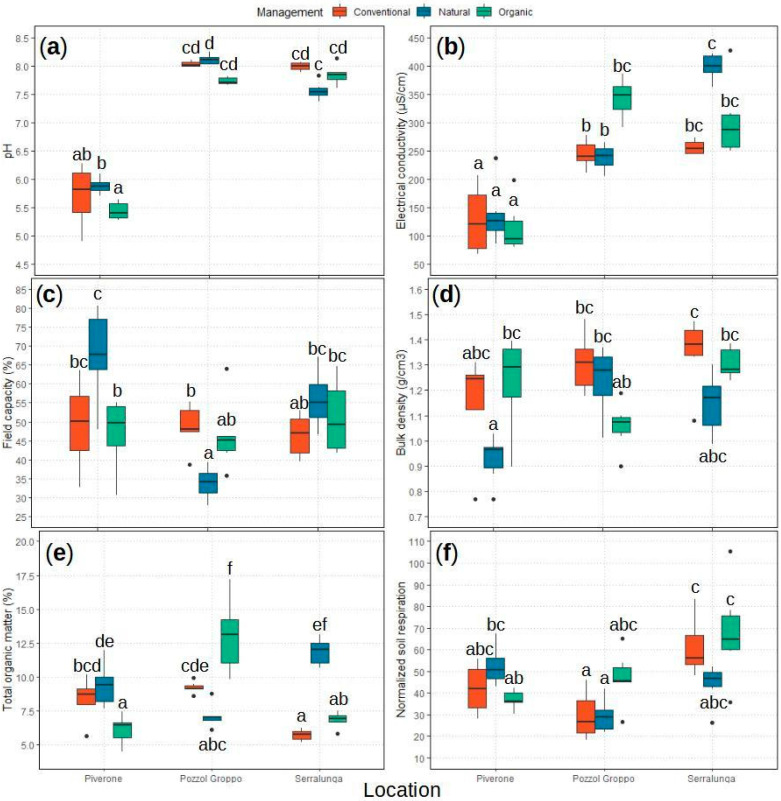
Boxplots of the main chemical and physical properties as well as the normalized soil respiration (nSR) of the topsoil horizons (0–5 cm) of studied soils for each location and management combination, (**a**) pH, (**b**) electrical conductivity (µS/cm), (**c**) field capacity (%), (**d**) bulk density (g/cm^3^), (**e**) total organic matter (%), and (**f**) normalized soil respiration (mg of soil organic carbon/100 g of dry soil). Different letters indicate statistically different comparisons based on post hoc tests with a “Tukey” correction. Horizontal line = median value; box = 25th–75th percentiles; whiskers = minimum and maximum values; dots = outliers.

We characterized the main chemical and physical properties (pH, electrical conductivity—EC, field capacity, total organic matter—TOM, and bulk density) of the topsoil horizons for each location and management combination (Figure 1). The location (L subscript) consisted of three areas: Pozzol Groppo (G_L_), Piverone (P_L_), and Serralunga (S_L_). The management (M subscript) included three separate environmental conditions: conventional vineyards (C_M_), organic vineyards (O_M_), and natural areas (N_M_).

Every studied soil property was significantly affected by the location x management interaction (pH: ANOVA(GLM): χ^2^_4,52_ = 28.807, *p* < 0.001; EC: ANOVA(GLM): χ^2^_4,52_ = 14.857, *p* = 0.005; field capacity: ANOVA(GLM): χ^2^_4,52_ = 40.643, *p* < 0.001; TOM: ANOVA(GLM): χ^2^_4,52_ = 449.613, *p* < 0.001; and bulk density: ANOVA(GLM): χ^2^_4,52_ = 18.494, *p* < 0.001; Table S1; Figure 1 and Figure S2). By performing post hoc pairwise comparisons on a model using a location × management variable, we confirmed that Piverone (P_L_) soil can be considered acidic while the soil collected in Pozzol Groppo (G_L_) and Serralunga (S_L_) soil were basic (see Figure 1a; Table S2A). The P_L_ EC was lower than the G_L_ and S_L_ EC (see Figure 1b, Table S2B). None of the other soil properties differed between locations across managements (see Figure 1c–e; Table S2C–E) and none of them showed differences between managements across locations (see Figure 1a–e; Table S2A–E). Details about other post hoc pairwise comparisons, such as management-related differences across locations, are reported in Table S2A–E and visible in Figure 1a–e.

In the presence of *L. paralienus* nests, the soil pH (ANOVA(GLM): χ^2^_1,52_ = 8.569, *p* = 0.003; Table S1) and EC increased (ANOVA(GLM): χ^2^_1,52_ = 4.755, *p* = 0.030; Table S1), while the bulk density decreased (ANOVA(GLM): χ^2^_1,52_ = 7.369, *p* = 0.006; Table S1).

#### 2.1.2. Soil Respiration: bSR and nSR

The total amount of C-CO_2_ efflux or basal soil respiration (bSR) was influenced by the location (ANOVA(GLM): χ^2^_2,52_ = 6.990, *p* = 0.031; Figure S3; Table S1) and the location x management interaction (ANOVA(GLM): χ^2^_4,52_ = 34.484, *p* < 0.001; Figure S3; Tables S1 and S2G). The normalized soil respiration (nSR), which was calculated from the bSR, was influenced by the location (ANOVA(GLM): χ^2^_2,52_ = 24.081, *p* < 0.001; Figure 1f; Table S1), and the location x management interaction (ANOVA(GLM): χ^2^_4,52_ = 16.270, *p* = 0.003; Figure 1f; Table S1). In detail, when a *L. paralienus* nest is present, the nSR increased significantly (ANOVA(GLM): χ^2^_1,52_ = 4.924, *p* = 0.026; Table S1). Details about other post hoc pairwise comparisons are reported in Table S2F,G and reported in Figure 1f and Figure S3.

### 2.2. Lasius Paralienus Cuticular Hydrocarbon Profile Description and Comparisons

**Figure 2:**
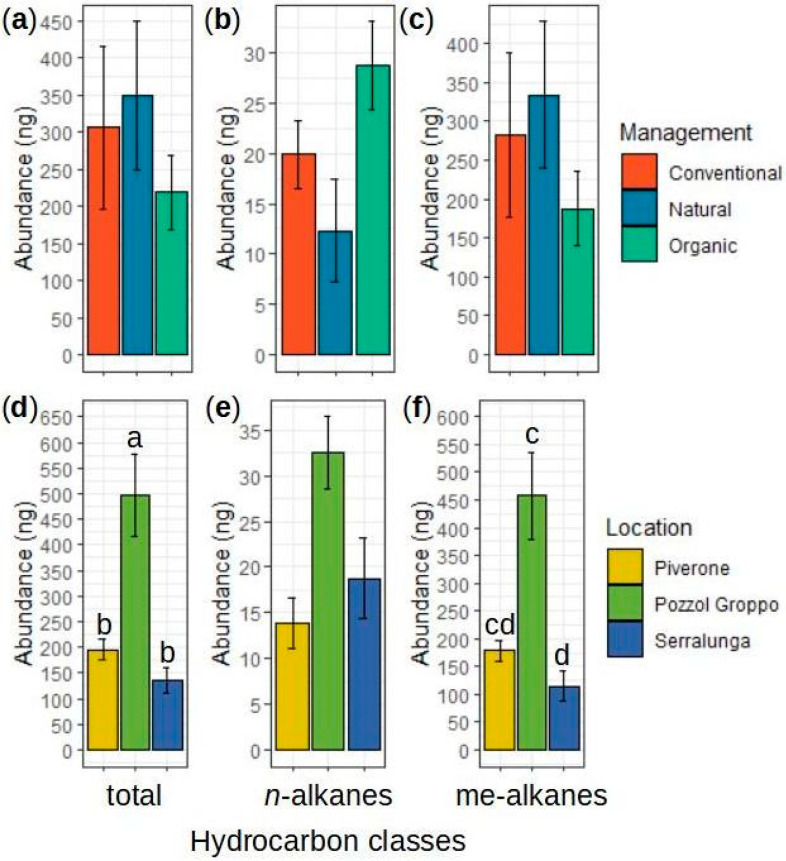
Abundance (±SE) of the CHC profile extracted from the body surface of *L. paralienus* workers—(**a**) total, (**b**) alkanes, and (**c**) methyl-branched alkanes—inhabiting zones subject to different management (natural area and conventional and organic vineyards); (**d**) total, (**e**) alkanes, and (**f**) methyl-branched alkanes collected from several locations (Piverone, Pozzol Groppo, Serralunga). Bars with different letters are statistically different according to GLM pairwise comparisons.

Overall, 135 CHCs distributed within 49 peaks were identified on the *Lasius paralienus* cuticle. The *L. paralienus* CHC profile is primarily composed of methylated linear alkane compounds (see Figure 2; Table 1). The identified CHCs are homologous series of linear alkanes, methyl-branched alkanes, and linear alkenes, between *n*-C_28_ and 13,23-diMeC_37_ (Table 2 and Table S3). Most of the colonies possessed all the analyzed CHC peaks but their abundances and relative proportions varied depending on the location and management.

#### 2.2.1. Univariate Analyses on the CHC Profile Variation

The management did not affect the overall CHC profile abundance (ANOVA(GLM): χ^2^_2,3_ = 1.473, *p* = 0.479; Table S4). By studying chemical compound classes separately, the management did not influence the abundance of methyl-branched alkanes (ANOVA(GLM): χ^2^_2,3_ = 1.934, *p* = 0.380; Table S4), but it affected the abundance of linear alkanes (ANOVA(GLM): χ^2^_2,3_ = 6.459, *p* = 0.040; Table S4). Nevertheless, none of the pairwise comparisons were significant (see Figure 2a–c; Table S4). Details on the analyses performed for each methylated compound class are reported in Table S4.

On the other hand, we pointed out a significant effect of the location on the overall CHC abundance (ANOVA(GLM): χ^2^_2,3_ = 34.257, *p* < 0.001; Table S4) with Pozzol Groppo (G_L_) ants’ profiles possessing a higher abundance of compounds than both Serralunga (S_L_) (Tukey: t = 2.687, *p* = 0.036; Figure 2d; Table S4) and Piverone (P_L_) (Tukey: t = 2.687, *p* = 0.036; Figure 2d; Table S4). By studying chemical compound classes separately, we found that the location also significantly affected the abundance of the linear alkane (ANOVA(GLM): χ^2^_2,3_ = 7.694, *p* = 0.021; Table S4) and of the methyl-branched alkanes (ANOVA(GLM): χ^2^_2,3_ = 27.419, *p* < 0.001; Table S4). By looking at the pairwise comparisons, we found G_L_ samples had a significantly higher methyl-branched alkanes’ abundance than S_L_ (Tukey: t = −4.015, *p* = 0.002; Figure 2f; Table S4). None of the other pairwise comparisons were significant (see Figure 2d–f; Table S4). Details on the analyses performed for each methylated compound class are reported in Table S4.

**Figure 3:**
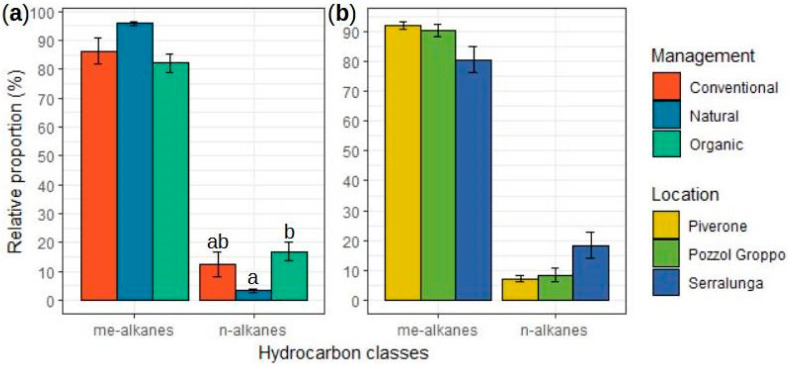
Relative proportions (±SE) of different CHC classes (alkanes and branched alkanes) extracted from the body surface of *L. paralienus* workers from—(**a**) several managements (natural area and conventional and organic vineyards) and (**b**) several locations (Piverone, Pozzol Groppo, Serralunga) colonies. Bars with different letters are statistically different according to GLM pairwise comparisons.

The relative proportion of linear alkanes was significantly impacted by both the location (ANOVA(GLM): χ^2^_2,3_ = 8.750, *p* = 0.013; Table S5) and the management (ANOVA(GLM): χ^2^_2,3_ = 14.236, *p* < 0.001; Table S5). Ants inhabiting organic vineyards (O_M_) exhibited a significantly higher relative proportion of linear alkanes than samples gathered from uncultivated areas (N_M_) (Tukey: t = 2.543, *p* = 0.049; Figure 3a; Table S5). None of the other pairwise comparisons were significant (see Figure 3a,b; Table S5). On the contrary, neither the location (ANOVA(GLM): χ^2^2,3 = 5.640, *p* = 0.060; Figure 3b; Table S5) nor the management (ANOVA(GLM): χ^2^2,3 = 5.852, *p* = 0.054; Figure 3a; Table S5) significantly impacted the relative proportion of methyl-branched alkanes. Details on the analyses performed for each methylated compound class are reported in Table S5.

We also performed separate GLMs on individual CHC peak abundance for location and management. The abundance of 45 CHC peaks out of 49 (Peaks: all of them except 1, 2, 9, and 15) was significantly impacted by the location (Table S6). By looking at the pairwise comparisons, 39 peaks (all of them except 1, 2, 5, 9, 12, 13, 15, 28, 46, and 48) had a higher abundance in Pozzol Groppo (G_L_) ants’ profile compared to Serralunga (S_L_) (Table S6), four peaks (12, 23, 27, and 33) had a higher abundance in G_L_ compared to Piverone (P_L_) (Table S6) and Peak 8 (15-, 14-, 13-, 12-, 11-MeC_30_+9,12-diMeC_30_) had a higher abundance in P_L_ compared to S_L_ (Tukey: t = −2.894, *p* = 0.023; Table S6). Only one peak (Peak 13: 4,8,14-triMeC_30_) was impacted significantly by management (ANOVA(GLM): χ^2^_2,3_ = 13.513, *p* = 0.001; Table S6), with its abundance being higher in the chemical profiles of ants collected in natural areas (N_M_) compared to those occurring in organic vineyards (O_M_) (Tukey: t = −2.826, *p* = 0.027; Table S6). Details about individual peak identification are reported in Table 2.

#### 2.2.2. Multivariate Analyses on the CHC Profile

**Figure 4:**
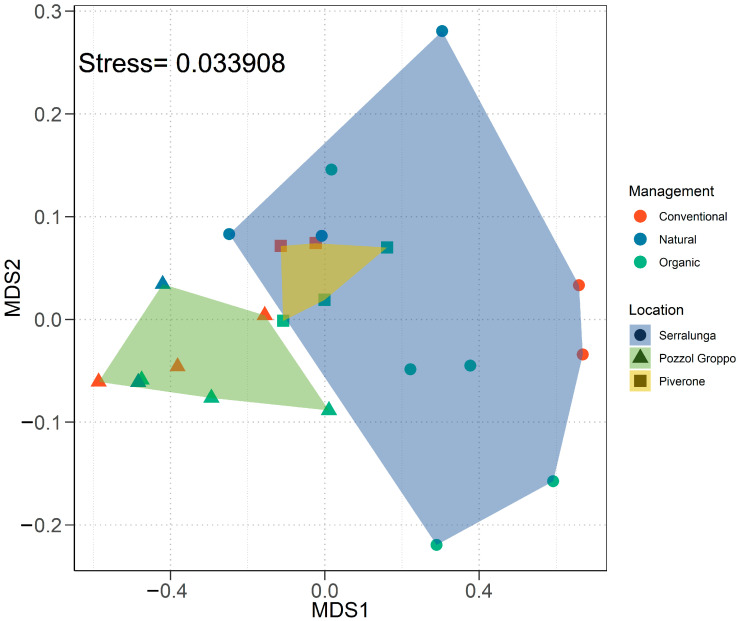
Non-metric multidimensional scaling plots (k = 2) of the CHCs extracted from *L. paralienus* workers depending on both location and management (based on the first and second MDS vectors).

The PERMANOVA test on the overall CHC profile confirmed that the management (PERMANOVA, F_2,23_ = 3.750, *p* = 0.026; Figure 4; Table S7) and the location (PERMANOVA, F_2,23_ = 15.488, *p* < 0.001; Figure 4; Table S7) had an influence on the overall *L. paralienus* CHC profile while their interaction was not significant (PERMANOVA, F_3,23_ = 1.898, *p* = 0.130; Table S7). The related pairwise comparisons revealed that the overall CHC profiles of workers from Pozzol Groppo (G_L_) were different from those of ants from both Piverone (P_L_) (PERMANOVA, F_1,13_ = 10.376, *p* = 0.006; Table S7) and Serralunga (S_L_) (PERMANOVA, F_1,18_ = 17.515, *p* < 0.001; Table S7). None of the pairwise comparisons among management types were significant (see Table S7 for statistics). When considering only the linear alkane fraction of CHC profiles, both the management (PERMANOVA, F_2,23_ = 10.367, *p* < 0.001; Table S7) and the location (PERMANOVA, F_2,23_ = 7.239, *p* = 0.002; Table S7) appeared to have a significant impact on it, but not their interaction (PERMANOVA, F_3,23_ = 1.026, *p* = 0.409; Table S7). The related pairwise comparisons revealed that the linear alkane CHC profiles of workers from G_L_ were different from those ants from both P_L_ (PERMANOVA, F_1,13_ = 10.155, *p* = 0.009; Table S7) and S_L_ (PERMANOVA, F_1,18_ = 5.067, *p* = 0.023; Table S7). In addition, the linear alkane CHC profiles differed between workers occurring in organic vineyards (O_M_) and natural areas (N_M_) (PERMANOVA, F_1,16_ = 7.398, *p* = 0.010; Table S7). The overall methyl-branched alkane fraction of the CHC profile was impacted by both the management (PERMANOVA, F_2,23_ = 3.432, *p* = 0.039; Table S7) and the location (PERMANOVA, F_2,23_ = 16.292, *p* < 0.001; Table S7) but their interaction had no significant effect (PERMANOVA, F_3,23_ = 1.980, *p* = 0.130; Table S7). The related pairwise comparisons revealed that the methyl-branched alkane CHC profiles of workers from G_L_ were different from those ants from both P_L_ (PERMANOVA, F_1,13_ = 9.708, *p* = 0.009; Table S7) and S_L_ (PERMANOVA, F_1,18_ = 18.743, *p* < 0.001; Table S7), while the methyl-branched alkane CHC profiles of workers from P_L_ were different from those ants from S_L_ (PERMANOVA, F_1,15_ = 4.200, *p* = 0.042; Table S7). None of the pairwise comparisons on the overall methyl-branched alkane CHC profiles among management types were significant (see Table S7 for statistics). Details on the analysis performed for each methylated compound class are reported in Table S7.

We used Indicator Species Analyses (ISA) on the overall CHC profile to determine whether some CHC peaks were significantly associated with one or more locations and/or management. To delve into the contribution of each compound to the dissimilarity of CHC assemblages, we also performed similarity percentages (SIMPER) analyses. Details about individual peak identification are presented in Table 2.

As for the location, the ISA showed that 45 peaks were at least partially associated with the Pozzol Groppo (G_L_) site, 40 of them (Peaks: all of them except 1, 2, 9, 15, 29, 32, 35, 41, and 42; Table S8) were only associated with G_L_, while 5 of them (Peaks: 29, 32, 35, 41, and 42; Table S8) were associated with the group G_L_+Piverone (P_L_). These results were corroborated by the SIMPER analyses, which detected 45 CHC peaks which were significantly affecting the dissimilarity between the G_L_ and Serralunga (S_L_) CHC profiles (G_L_-S_L_: all the peaks were significant except Peak 1 (47th average), *n*-C_28_, *p* = 0.054; Peak 2 (29th average), *n*-C_29_, *p* = 0.418; Peak 12 (39th average), *n*-C_31_, *p* = 0.675; and Peak 15 (34th average), 5-MeC_31_, *p* = 0.863; Supplementary Table S9A) with a higher abundance in G_L_ than in S_L_. The “average” rank used above corresponds to the “average” contribution of this CHC peak to the average dissimilarity between the CHC profiles from the two compared groups. Two CHC peaks significantly affected the dissimilarity between G_L_ and P_L_ CHC profiles. One peak (G_L_-P_L_: Peak 39 (8th average), 6,16-, 6,14-, 6,12-diMeC_34_, *p* = 0.042; Table S9B) had a higher abundance in G_L_ than in P_L_ while another peak (G_L_-P_L_: Peak 23 (23rd average), 6-MeC_32_, *p* = 0.012; Table S9B) had a lower abundance in G_L_ than in P_L_. For the last location contrast, one peak (P_L_-S_L_: Peak 32 (1st average), 7,25-, 7,21-, 7,19-, 7,17-diMeC_33_, *p* = 0.031; Table S9C) significantly affected the dissimilarity between P_L_ and S_L_ CHC profiles with a higher abundance in P_L_ than in S_L_.

Regarding the management, the ISA showed that only Peak 1 (*n*-C_28_) was associated with the chemical profile of samples collected in organic vineyards(O_M_) (Stat = 0.555, *p* = 0.027; Table S10), and Peak 2 (*n*-C_29_) was associated with the group O_M_+conventional vineyards (C_M_) (Stat = 0.517, *p* = 0.049; Table S10). The SIMPER analyses highlighted that two CHC peaks were significantly affecting the dissimilarity between O_M_ and C_M_ CHC profiles (O_M_-C_M_: Peak 3 (48th average), 15-, 13-, 11-MeC_29_, *p* = 0.040; Peak 14 (19th average), 15-, 13-, 11-MeC_31_, *p* < 0.05; Table S11A) with a lower abundance in O_M_ in contrast to C_M_. Four CHC peaks significantly affected the dissimilarity between the O_M_ and natural areas (N_M_) CHC profiles. Three CHC peaks (O_M_-N_M_: Peak 2 (1st average), *n*-C_29_, *p* = 0.005; Peak 6 (36th average), *n*-C_30_, *p* = 0.005; and Peak 12 (15th average), *n*-C_31_, *p* = 0.028; Table S11B) had a higher abundance in O_M_ than in N_M_, while only one peak (O_M_-N_M_: Peak 13 (23rd average), 4,8,14-triMeC_30_, *p* = 0.020; Table S11B) had a lower abundance in O_M_ than in N_M_. Finally, the same peak significantly affected the dissimilarity between C_M_ and N_M_ CHC profiles (C_M_-N_M_: Peak 13 (29th average), 4,8,14-triMeC_30_, *p* = 0.044; Table S11C) with a lower abundance in C_M_ in contrast to N_M_.

## 3. Discussion

The chemical profile of *Lasius paralienus* has never been described before. With 135 identified CHCs between *n*-C_28_ and 13,23-diMeC_37_, *L. paralienus* encompasses a richer amount of compounds than co-occurring common ants, such as *Myrmica* species (on average 37–46 CHCs, see [44] and [45], respectively). In contrast, the *L. paralienus* CHC assembly is similar to the chemical signature of the congeneric *Lasius niger*, which possesses around 109 CHCs between *n*-C_25_ and 11,15-diMeC_37_ [46]. The two species of the genus *Lasius* share several compounds, but at least 30 are found only in *L. paralienus* [46].

A multitude of factors impact CHC profiles, presenting a challenge in disentangling their effects [21]. We performed multivariate analyses to achieve a comprehensive understanding of the variation within CHC composition, and we used a univariate approach to assess CHC traits, such as proportions of CHC classes, the number of homologous series, and variation within distinct CHC classes, to provide insights into the effects of location or management on the specific function of compounds, like waterproofing or communication. Moreover, we accounted for the soil characteristics as a proxy for microhabitat variations.

Significant variations in the CHC profiles among areas scattered on geographical gradients [45] or altitudes [47] have been previously observed in other ant species, while studies on environmental changes due to human activities are entirely lacking. Indeed, our findings revealed that location is the most crucial factor affecting the *L. paralienus* CHC profile quantitatively, while no differences in the CHC assemblages were observed. Contrary to our predictions, this effect was more important than the anthropic impact, related to various levels of management. In detail, ants sampled from the Pozzol Groppo area possessed significantly different CHC profiles from those collected in Piverone and Serralunga, with a higher overall CHC abundance. More precisely, the Pozzol Groppo area was especially different from Serralunga, with higher differences between these two areas compared to the differences between Pozzol Groppo and Piverone or between Piverone and Serralunga. Soil properties partially explained these area-related differences in chemical profiles as reported by previous studies, which found that local diet and soil conditions quantitatively affected the colony’s CHC blends [48]. As expected, pH, EC, field capacity, bulk density, TOM, bSR, and nSR, which are linked to environmental or land-use differences [49], varied significantly across location and management combinations (see Figure 1a–f and Figure S3). However, the homogenization of some soil properties such as pH, EC, or nSR (see Figure 1a,b,f) indicates that irrespective of the management, specific soil features are more similar in fields of the same location. Among those, normalized soil respiration is a proxy for soil microbial activity [50,51], and the microorganism community potentially affects the ant CHC profile [52,53]. Indeed, changes in the ants’ external microbiota can influence nestmate recognition [54]. This potential soil microbial influence on the CHC profile is supported by our results, primarily highlighting differences in nSR between Pozzol Groppo and Serralunga locations (see Figure 1f). Moreover, the inconsistent impacts of management our data suggest may be moderated more directly by how the microbial community interacts with the location-specific environmental variables while under those management methods [55]. Although details are unknown, the soil can indirectly affect colony odors in several ways, as it can be used as a vector to transfer CHC between colony members [56]. Vice versa, we also found that the presence of *L. paralienus* nests significantly affected some vineyard properties, such as pH, EC, bulk density, and normalized soil respiration. When studying *Lasius niger*, Frouz and colleagues [57] observed a modification of the pH and several other chemical properties related to the nest presence, whereas soil respiration in previous studies on *Lasius niger* and *Lasius flavus* revealed more contrasting results with rather an absence of nest impact [58]. In addition, ant nest-mediated changes in soil properties may also be associated with changes in the microbial communities [59,60,61]. Therefore, it is not straightforward to disentangle the exact causal relation between soil features and ant colony traits as these are just two of the many factors occurring in these complex agroecosystems, and soil microorganisms could act as pivotal drivers of environmental change.

The analysis of the *L. paralienus* chemical profile pointed out several CHC peaks that were associated with a location or concurred to strengthen the dissimilarity between two sites. Only the abundances of *n*-C_28_, *n*-C_29_, and 5-MeC_31_ CHCs were not area-related, while 4-MeC_30_ and 7,14-diMeC_30_ only significantly affected the dissimilarity between the G_L_ and S_L_ CHC profiles. It is interesting to note that *n*-C_28_ and *n*-C_29_ are commonly found in several species’ CHC profiles; thus, they can be associated with some pivotal functional roles [46,62,63,64].

The influence of distinct managements on CHC assemblages was less pronounced than expected. Indeed, the multivariate analyses revealed that agricultural practices had an effect on the overall profile, but primarily on the linear alkanes rather than methylated alkanes. Among the latter, monomethylated alkanes were the compounds most affected by the management (see Table S7 for statistics). The structural class affects the CHC biophysical properties and, thus, their functional role in the cuticle [65]. Linear and terminally branched monomethyl alkanes possess a better water loss prevention capability because of their structure, which allows the formation of more viscous or solid barriers that are more stable under temperature changes [33,66,67,68]. Therefore, the presence of *n-*alkanes is an essential environmental adaptation that prevents desiccation [69,70]. In contrast, the position of methyl or double bonds may render compounds more informative for social insect recognition than simple modifications of the CHC chain length in alkanes [14,71,72]. Moreover, dimethylalkanes are used in the nestmate recognition system as more precise discrimination signals than monomethylalkanes [14]. Still, the knowledge of CHC class functions is far from being fully disentangled, and neither the methyl-branched role in insect desiccation resistance [70,73] nor the linear alkanes contribution in chemical communication can be ruled out [74]. Our results showed that differences between the chemical profiles of ants occurring in vineyards with respect to close natural areas are mainly driven by *n*-C_28_ and *n*-C_29_ (linear alkanes), which are associated with the vineyards’ management and 4,8,14-triMeC_30_ which is significantly more abundant in natural areas than in vineyards. The less-marked differences between the two vineyard management types were primarily caused by the higher abundance of 15-MeC_29_, 13-MeC_29_, 11-MeC_29_, 15-MeC_31_, 13-MeC_31_, and 11-MeC_31_ in conventional than organic fields. At least for the 11-MeC_29_, there is evidence that this methyl-branched alkane is a key compound for *Polistes dominulus* nestmate recognition [75].

Although our results pointed out a stronger effect of locations on *L. paralienus* CHC variations, the management also affected the abundance of some compounds that can be pivotal for maintaining the colony identity. If recognition systems are disrupted by human activity, this will negatively impact crucial ants’ ecosystem services, eventually affecting environmental sustainability [76]. Because of the scanty knowledge of each compound’s or class of compounds’ functions, behavioral tests performed by manipulating the concentration or presence of single compounds are necessary to reach conclusive remarks.

## 4. Materials and Methods

### 4.1. Sample Collection

We sampled distinct colonies of *Lasius paralienus* in three locations 75 km apart from each other (Piverone (P_L_), Italy, 45°26′51″ N, 8°0′25″ E, annual pluviosity: 1396 mm, annual mean temperature: 12.58 °C; Pozzol Groppo (G_L_), Italy, 44°52′39″ N, 9°1′45″ E, annual pluviosity: 836 mm, annual mean temperature: 14.67 °C; Serralunga d’Alba (S_L_), Italy, 44°36′40″ N, 8°0′2″ E, annual pluviosity: 1080 mm, annual mean temperature: 12.83 °C–see Supplementary Materials Figure S1). For each location, ants were sampled at two different vineyards under diverse management, three colonies at each conventional (C_M_) and organic vineyards (O_M_) and three ant colonies were also collected from an unmanaged field, hereafter called the “natural” area (N_M_). Just at the organic vineyard in Serralunga, we collected 5 nests. This sampling led to a total of 29 colonies collected for further CHC analysis. Ants were sampled within a week, from unparasitized nests far from aphid communities to reduce the number of variables affecting CHC profile variations. *Lasius paralienus* was identified morphologically using the identification key by Seifert [37].

To control for microhabitat variations, we also sampled soil directly from *L. paralienus* nests and in their vicinity (one meter away) following a modified protocol by Blanco-Moure, Nuria, et al. [77] focused on the first five centimeters of the soil where the *L. paralienus* nests were present. In detail, metallic cylinders (98.17 cm^3^) were used to collect 4 samples of 0–5 cm depth within a circle of a 15 cm radius. These 4 subsamples were mixed together to form one composite soil sample. Three *L. paralienus* nests were marked for each location x management combination. For each nest, one composite soil sampling made of 4 subsamples was performed inside the nest and another one at one meter of the nest was used as a control. For each location x management combination 6 composite soil samples were sampled, 3 inside a *L. paralienus* nest and 3 outside, and this sampling led to a total of 54 soil samples (3 nests × 3 management × 3 locations = 27 gathered from ant nests and 27 outside).

### 4.2. Chemical Analyses

Extractions were performed the day after the colony samplings. For each colony, 5 individuals were pooled together in separate clean glass vials. The ants were frozen for one hour at −20 °C before the extraction. The CHCs were extracted by immersion in 200 µL of HPLC-grade hexane (chemicals from CARLO ERBA Reagents S.r.l., Cornaredo, Italy) for 10 min under agitation. The solvent was moved to another clean glass vial containing an insert and was evaporated at room temperature in a laminar flow cupboard. After drying, the extract was stored at −20 °C until analysis. The dry extract was dissolved in 20 µL of an HPLC-grade pentane solution (chemicals from CARLO ERBA Reagents S.r.l., Cornaredo, Italy) for the chemical analysis. The pentane contained Tetradecane (C_14_H_30_/CH_3_(CH_2_)_12_CH_3_) as an internal standard at the concentration of 2.5 ng/µL. In total, 2 µL of this solution was injected in an Agilent Technologies 7890A Gas-Chromatograph (Agilent Technologies, Santa Clara, CA, USA) coupled to an Agilent 5975C Mass Spectrometer (Agilent Technologies, Santa Clara, CA, USA) (GC-MS) using the “Agilent MSD Productivity ChemStation version E.02.01.1177” software as the system control software. The injector temperature was 280 °C, and we used an HP-5MS (5%-phenyl)-methylpolysiloxane phase capillary column (30 m × 0.25 mm × 0.25 µm) with helium as the carrier gas at 1 mL/min. Injection was splitless, and the oven temperature was held at 70 °C for 1 min, then increased to 210 °C at 30 °C/min, and then to 320 °C at 4 °C/min and held for 5 min. Mass spectra were recorded with electron impact ionization at 70 eV. The program lasted a total of 40 min. Between each sample analysis, the instrument cooled down for 15 min. We integrated the chromatograms using the “Agilent MSD ChemStation version E.02.01.1177’’ GC-MS software. Firstly, automatic integration was performed with the following parameters for the *Lasius paralienus* samples (Initial area reject: 0; Initial peak width: 0.017; Shoulder detection: OFF; Initial threshold 20.0). Then, we manually checked and corrected the automatic integration results. We calculated the absolute quantity (ng) of each sample CHC and applied a logarithmic transformation to this quantity (ln(quantity + 1)). We identified CHCs by using the ion fragmentation information. Because of contamination, one sample from the G_L_ natural area, and the S_L_, P_L_ conventional vineyard, and three from the P_L_ natural area were removed from the further statistical analyses.

### 4.3. Soil Samples Analyses

All soil samples were sieved on a 2 mm mesh and packed in plastic bags under refrigeration (4 °C) prior to further analyses. The methods described by Page et al. [78] were followed for several analytical determinations. Microbial activity was measured by capturing the CO_2_ emitted from the soil in NaOH traps on selected days during an incubation period of 96 days. During this incubation period, soil samples at half field capacity were kept in darkness at a temperature of 25 ± 1 °C; from this assay, we calculated the basal soil respiration (bSR) or C-CO_2_ efflux over this period as well as the normalized soil respiration (nSR) as the SR per oxidizable C unit and time, i.e., the rate of organic carbon mineralization [50]. Detailed protocols for the latter determinations have been extensively described in previous works [51,79,80].

The soil reaction was measured in a 1:2.5 soil-to-water ratio (pH 1:2.5), and the soil electrical conductivity in a 1:5 soil-to-water ratio (CE 1:5). The total organic matter (TOM) was determined by weight loss on ignition. The field capacity was obtained by comparing the weight of soil samples saturated with water after 24 h of draining by gravity and their weight after being dried at 105 °C until they reached a stable weight. This treatment enabled us to refer all the results to dry soil [78]. The bulk density was calculated by dividing each composite soil sample’s total weight by 4 times the metallic cylinder volume (392.68 cm^3^). Because of abnormal results, two soil samples from the P_L_-C_M_ sampled inside and at one meter of the same nest were removed from the further statistical analyses.

### 4.4. Statistical Analyses

Statistical analyses were performed with the software R v.4.3.0 using the R packages “stats,” “lme4,” “car,” “vegan,” “indicspecies,” “emmeans,” and “pairwiseAdonis.”

Generalized linear mixed-effects models (GLMMs) were performed with the pH, EC, field capacity, TOM, bulk density, bSR, or nSR as response variables, “presence or absence of *L. paralienus* nest,” “location,” “management,” and “location × management interaction” as fixed variables, and “nest number” as a random factor by using the “lmer” function (“lme4” package) for a Gaussian-distributed model and the “glmer” function (“lme4” package) for the gamma-distributed model, to assess the variations in soil properties.

Generalized linear models (GLMs) were performed with the pH, EC, field capacity, TOM, bulk density, bSR, or nSR as response variables and “zone” (the variable containing all the location × management combinations) as the fixed variable by using the “glm” function (“stats” package) for the gamma-distributed model, to assess the variations in soil properties across location × management combinations.

Generalized linear models (GLMs) were performed with the overall, linear alkane, methyl-branched alkane taken all together, monomethyl-branched alkane, dimethyl-branched alkane, and trimethyl-branched alkane CHCs’ absolute quantity or relative abundance as the response variable and “location” or “management” as fixed variables by using the “glm” function (“stats” package) to survey the variations in the *L. paralienus* chemical profile.

Generalized linear models (GLMs) were performed with the absolute quantity of each individual CHC peak as the response variable and “location” or “management” as fixed variables by using the “glm” function (“stats” package) to test for single CHC peak changes.

After testing the normality of the residuals of Gaussian-distributed models, comparing the data distribution to the gaussian and gamma distributions and comparing the Gaussian-distributed models’ and gamma-distributed models’ AIC and deviance, the gamma-distributed models were chosen for every response variable. An analysis of variance (ANOVA) of each model was performed to obtain the *p*-values by using the “ANOVA” function (“car” package). The individual CHC peaks’ results were corrected using the Benjamini and Hochberg method, given that we had conducted 49 separate tests for each variable. When necessary, post hoc tests were performed with a “Tukey” correction by using the “emmeans” function (“emmeans” package) on the models.

Permutational multivariate analyses of variance (PERMANOVA) using distance matrices were performed by using the “adonis2” function (“vegan” package) with a matrix containing the different CHC peaks’ log-transformed absolute quantities as the response matrix, “location,” “management,” and the “location × management interaction” as fixed variables, the Bray–Curtis method to calculate the pairwise distances, and 99,999 permutations without any permutation restriction. These tests were repeated by alternatively using a CHC matrix containing the overall CHC profile, only linear alkanes, only methyl-branched alkanes taken all together, only monomethyl-branched alkanes, only dimethyl-branched alkanes, or only trimethyl-branched alkanes. Pairwise comparisons of the previous statistical results were performed by using the “pairwise.adonis2” function (“pairwiseAdonis” package).

To assess which CHC peak was strongly associated with a specific location-management combination, multi-level pattern analyses were performed by using the “multipatt” function (“indicspecies” package) with a matrix containing the different CHC log-transformed absolute quantity as the response matrix, “location” or “management” as fixed variables, the group-equalized phi coefficient as the CHC-site group association function, and 99,999 permutations without any permutation restriction.

To assess which CHC peak was significantly impacting the dissimilarity between CHC profiles from either the “location” or “management” variables and the average contribution of this CHC peak to this average dissimilarity, similarity percentages (SIMPER) tests were performed by using the “simper” function (“vegan” package) with a matrix containing the CHC log-transformed absolute quantity as the response matrix, “location” or “management” as fixed variables, and 99,999 permutations without any permutation restriction.

## 5. Conclusions

The newly defined cuticular hydrocarbon profile of *L. paralienus* possesses a CHC assembly similar to *L. niger* but with species-specific compounds. Our results showed that *L. paralienus*’ CHC profile variations were more led by area changes than by management occurring in the agroecosystems, even if both were impactful. Two linear alkanes, *n*-C_28_ and *n*-C_29_, were tightly associated with vineyard management. Their presence in several species suggests their functional importance for desiccation resistance. In addition, the dissimilarities between CHC profiles, according to various managements, were also driven by a few methylated-branched alkanes, which can be pivotal for ants’ nestmate recognition abilities. The intricate relationships occurring in such complex agroecosystems among the geographical variables, management, soil properties, and colony traits of soil-modifying insects, such as ants, require extensive studies performed on a larger sample size and geographical range and likely including the investigation of the microbial community to be fully disentangled. However, our findings contributed to shed light on this system’s interactions by reporting non-negligible impacts of human activities on ant CHC profiles, which can disrupt a crucial ability of social insects, eventually affecting their role in the ecosystem service.

## Figures and Tables

**Table 1:** Classification of *Lasius paralienus* cuticular hydrocarbons depending on their compound class.

Profile Features	Linear Alkane	Linear Alkene	Methylated Linear Alkane			
			Monomethyl- Alkanes	Dimethyl- Alkanes	Trimethyl- Alkanes	Methylated Linear Alkane Total
CHC number	5	1	45	69	15	129
CHC compound class (%)	3.70	0.74	33.34	51.11	11.11	95.56
Number of peaks	4 ^1^	0 ^2^	11 ^3^	17 ^3^	9 ^3^	43 ^1,2,3^
Peak compound class (%)	8.16	0	22.45	34.69	18.37	87.76

^1^ One peak is a mix of linear alkane and methylated linear alkane and was thus not accounted for in this table. ^2^ One peak is a mix of linear alkene and methylated linear alkane and was thus not accounted for in this table. ^3^ Six peaks are a mix of several methylated linear alkane sub-compound classes and are thus only accounted for in the methylated linear alkane total category.

**Table 2:** Identification table of the main cuticular hydrocarbons of *Lasius paralienus* (peaks retained for analyses in the present study).

Peak Correspondence					
n	Peak Identified Compounds	n	Peak Identified Compounds	n	Peak Identified Compounds
1	*n*-C_28_	18	5,15-, 5,13-, 5,9-diMeC_31_	35	5,9,15-, 5,7,15-triMeC_33_
2	*n*-C_29_	19	7,11,15-triMeC_31_	36	17-, 16-, 15-, 14-, 13-, 12-MeC_34_
3	15-, 13-, 11-MeC_29_	20	5,9,15-triMeC_31_+ 5,7,15-, 5,7,13-, 5,7,11-triMeC_31_	37	8,16-diMeC_34_
4	3-MeC_29_	21	16-, 15-, 14-, 13-, 12-, 11-, 10-MeC_32_	38	8,12,16-triMeC_34_
5	5,15-, 5,13-, 5,9-diMeC_29_	22	9-MeC_32_+8,16-, 8,14-, 8,12-diMeC_32_	39	6,16-, 6,14-, 6,12-diMeC_34_
6	*n*-C_30_	23	6-MeC_32_	40	4,16-, 4,14-, 4,12-diMeC_34_
7	5,9,11-triMeC_29_	24	4-MeC_32_+8,12,15-triMeC_32_+8,16, 8,14-, 8,12-diMeC_32_	41	17-, 15-, 13-MeC_35_
8	15-, 14-, 13-, 12-, 11-MeC_30_+9,12-diMeC_30_	25	6,16-, 6,14-, 6,12-, 6,10-diMeC_32_+5,15-, 5,13-diMeC_32_	42	13,21-diMeC_35_
9	4-MeC_30_+7,14-diMeC_30_	26	4,16-, 4,14-, 4,12, 4,10-, 4,8-diMeC_32_	43	7,27-, 7,25-, 7,23-, 7,21-, 7,19-, 7,17-diMeC_35_
10	X-C_31:1_+5,15-diMeC_30_+6,12-diMeC_30_	27	*n*-C_33_+6,10,16-triMeC_32_	44	5,19-, 5,17-, 5,15-, 5,13, 5,9-diMeC_35_
11	4,14-, 4,12-, 4,10-, 4,8-diMeC_30_	28	4,8,16-triMeC_32_	45	5,9,15-triMeC_35_
12	*n*-C_31_	29	17-, 15-, 13-, 11-MeC_33_	46	8,18-diMeC_36_
13	4,8,14-triMeC_30_	30	7-MeC_33_	47	14,22-diMeC_36_+12,24-diMeC_36_
14	15-, 13-, 11-, 9-MeC_31_	31	5-MeC_33_+13,21-diMeC_33_+11,17-diMeC_33_+9,17-diMeC_33_	48	19-, 17-, 15-, 13-MeC_37_
15	5-MeC_31_	32	7,25-, 7,21-, 7,19-, 7,17-diMeC_33_	49	13,23-diMeC_37_
16	9,15-, 9,13-diMeC_31_	33	5,17-, 5,15-, 5,13-, 5,9-diMeC_33_		
17	7,23-, 7-19-diMeC_31_+3-MeC_31_	34	7,11,15-triMeC_33_		

## Data Availability

The raw data supporting the conclusions of this article are available at the following link https://doi.org/10.6084/m9.figshare.27103216.v1.

## Supplementary Materials

The supporting information can be downloaded at https://www.mdpi.com/article/10.3390/ijms251910407/s1.
