# Supplementary material for: Variation in Ants’ Chemical Recognition Signals across Vineyard Agroecosystems
Source: Int J Mol Sci. 2024 Sep 27;25(19):10407. doi: 10.3390/ijms251910407 (PMC11477430; doi:10.3390/ijms251910407)
Supplement: Supplementary file 1 [file ijms-25-10407-s001.zip › ijms-3195765-supplementary.pdf]

Supplementary material

Table S1. Anova results of the GLM models for location, management, presence of *Lasius paralienus* and location x management interaction effects on the soil main chemical, physical and biological properties of topsoil horizons (0–5 cm).

Properties	Variable	Test statistic (χ^2)	df	p-value
pH (H_2O)	Location	1479.03339	2	**6.76869934802508e-322*** **
	Management	16.0241275	2	**0.00033144*** **
	Presence of *L. paralienus*	8.56862051	1	**0.00342006** **
	Location x Management	28.8071527	4	**8.56E-06*** **
EC (dS m-1)	Location	137.70505	2	**1.25E-30*** **
	Management	4.96596122	2	0.08349399
	Presence of *L. paralienus*	4.75500262	1	**0.02921325* **
	Location x Management	14.8570608	4	**0.00500705** **
Field capacity (%)	Location	23.4160653	2	**8.23E-06*** **
	Management	0.80368153	2	0.66908728
	Presence of *L. paralienus*	3.4483737	1	0.06331404
	Location x Management	40.6432356	4	**3.19E-08*** **
TOM (%)	Location	43.6402754	2	**3.34E-10*** **
	Management	28.2066684	2	**7.50E-07*** **
	Presence of *L. paralienus*	0.73361381	1	0.39171431
	Location x Management	449.613426	4	**5.27E-96*** **

Bulk density ($g.cm^{-3}$)	Location	11.0280283	2	**0.0040299****
Bulk density ($g.cm^{-3}$)	Management	10.5682872	2	**0.00507137****
Bulk density ($g.cm^{-3}$)	Presence of *L. paralienus*	7.36931157	1	**0.00663463****
Bulk density ($g.cm^{-3}$)	Location x Management	18.4935993	4	**0.00098799*****
C-CO2 efflux (mg of CO2 per 100 g of dry soil)	Location	6.98955111	2	**0.03035556***
C-CO2 efflux (mg of CO2 per 100 g of dry soil)	Management	3.60469381	2	0.1649114
C-CO2 efflux (mg of CO2 per 100 g of dry soil)	Presence of *L. paralienus*	3.0639159	1	0.08004854
C-CO2 efflux (mg of CO2 per 100 g of dry soil)	Location x Management	34.4841279	4	**5.93E-07*****
Normalized Soil respiration (mg of Soil Organic Carbon per 100 g of dry soil)	Location	24.0814012	2	**5.90E-06*****
Normalized Soil respiration (mg of Soil Organic Carbon per 100 g of dry soil)	Management	3.81197684	2	0.14867562
Normalized Soil respiration (mg of Soil Organic Carbon per 100 g of dry soil)	Presence of *L. paralienus*	4.9240515	1	**0.02648534***
Normalized Soil respiration (mg of Soil Organic Carbon per 100 g of dry soil)	Location x Management	16.2702974	4	**0.00267711****

*/**/***: Asterisks denote significant p-values (p: ***<0.001<**<0.01<*<0.05)

Table S2.A Tukey pairwise comparison results of the GLM model for the location x management interaction effect on the pH variable for the topsoil horizon (0-5 cm).

**Contrast**	**Estimate**	**SE**	**df**	**t ratio**	**p value**
GL;OM - GL;CM	-0.0382832	0.01913682	43	-2.00049933	0.55139162
GL;OM - GL;NM	-0.04778348	0.01913682	43	-2.49693903	0.26194218
GL;OM - PL;OM	0.35194273	0.01913682	43	18.3908667	**1.10E-12*** **
GL;OM - PL;CM	0.30420419	0.02139562	43	14.218061	**1.10E-12*** **
GL;OM - PL;NM	0.27298537	0.01913682	43	14.2649272	**1.10E-12*** **
GL;OM - SL;OM	-0.01434253	0.01913682	43	-0.74947316	0.99763703
GL;OM - SL;CM	-0.03245592	0.01913682	43	-1.69599319	0.74550903
GL;OM - SL;NM	0.02201724	0.01913682	43	1.15051698	0.96239658
GL;CM - GL;NM	-0.00950028	0.01913682	43	-0.4964397	0.99988289
GL;CM - PL;OM	0.39022593	0.01913682	43	20.391366	**1.10E-12*** **
GL;CM - PL;CM	0.34248739	0.02139562	43	16.007362	**1.10E-12*** **
GL;CM - PL;NM	0.31126857	0.01913682	43	16.2654266	**1.10E-12*** **
GL;CM - SL;OM	0.02394066	0.01913682	43	1.25102617	0.93973372
GL;CM - SL;CM	0.00582728	0.01913682	43	0.30450614	0.99999729
GL;CM - SL;NM	0.06030044	0.01913682	43	3.15101631	0.06591634
GL;NM - PL;OM	0.39972621	0.01913682	43	20.8878057	**1.10E-12*** **
GL;NM - PL;CM	0.35198766	0.02139562	43	16.4513912	**1.10E-12*** **
GL;NM - PL;NM	0.32076884	0.01913682	43	16.7618663	**1.10E-12*** **
GL;NM - SL;OM	0.03344094	0.01913682	43	1.74746587	0.71455535
GL;NM - SL;CM	0.01532756	0.01913682	43	0.80094584	0.996259
GL;NM - SL;NM	0.06980071	0.01913682	43	3.64745601	**0.01846922***
PL;OM - PL;CM	-0.04773855	0.02139562	43	-2.23123022	0.40491489
PL;OM - PL;NM	-0.07895737	0.01913682	43	-4.12593943	**0.00475601*** *
PL;OM - SL;OM	-0.36628527	0.01913682	43	-19.1403398	**1.10E-12*** **
PL;OM - SL;CM	-0.38439865	0.01913682	43	-20.0868599	**1.10E-12*** **
PL;OM - SL;NM	-0.3299255	0.01913682	43	-17.2403497	**1.10E-12*** **
PL;CM - PL;NM	-0.03121882	0.02139562	43	-1.45912219	0.86762186

$P_L;C_M$ - $S_L;O_M$	-0.31854672	0.02139562	43	-14.8884102	**1.10E-12*** **
$P_L;C_M$ - $S_L;C_M$	-0.33666011	0.02139562	43	-15.7350034	**1.10E-12*** **
$P_L;C_M$ - $S_L;N_M$	-0.28218695	0.02139562	43	-13.1890073	**1.11E-12*** **
$P_L;N_M$ - $S_L;O_M$	-0.2873279	0.01913682	43	-15.0144004	**1.10E-12*** **
$P_L;N_M$ - $S_L;C_M$	-0.30544129	0.01913682	43	-15.9609204	**1.10E-12*** **
$P_L;N_M$ - $S_L;N_M$	-0.25096813	0.01913682	43	-13.1144103	**1.11E-12*** **
$S_L;O_M$ - $S_L;C_M$	-0.01811338	0.01913682	43	-0.94652002	0.98863831
$S_L;O_M$ - $S_L;N_M$	0.03635977	0.01913682	43	1.89999014	0.6173984
$S_L;C_M$ - $S_L;N_M$	0.05447316	0.01913682	43	2.84651016	0.13156271

*/**/***: Asterisks denote significant p-values (p: ***<0.001<**<0.01<*<0.05)

Table S2.B Tukey pairwise comparison results of the GLM model for the location x management interaction effect on the EC variable for the topsoil horizon (0-5 cm).

Contrast	Estimate	SE	df	t ratio	p value
GL;OM - GL;CM	0.33845845	0.1448681	43	2.33632144	0.34419882
GL;OM - GL;NM	0.36353489	0.1448681	43	2.50941983	0.25613317
GL;OM - PL;OM	1.09424483	0.1448681	43	7.55338701	**7.15E-08*** **
GL;OM - PL;CM	0.97684924	0.16196746	43	6.03114502	**1.11E-05*** **
GL;OM - PL;NM	0.91408036	0.1448681	43	6.3097421	**4.39E-06*** **
GL;OM - SL;OM	0.1226277	0.1448681	43	0.84647825	0.99455532
GL;OM - SL;CM	0.29219895	0.1448681	43	2.01699995	0.54056806
GL;OM - SL;NM	-0.15127022	0.1448681	43	-1.04419272	0.97892491
GL;CM - GL;NM	0.02507644	0.1448681	43	0.17309839	0.99999997
GL;CM - PL;OM	0.75578638	0.1448681	43	5.21706557	**0.00015993*** **
GL;CM - PL;CM	0.63839079	0.16196746	43	3.94147559	**0.00812599** **
GL;CM - PL;NM	0.57562191	0.1448681	43	3.97342066	**0.00741387** **
GL;CM - SL;OM	-0.21583075	0.1448681	43	-1.48984319	0.85402639
GL;CM - SL;CM	-0.0462595	0.1448681	43	-0.31932149	0.99999607
GL;CM - SL;NM	-0.48972867	0.1448681	43	-3.38051416	**0.03734699***
GL;NM - PL;OM	0.73070995	0.1448681	43	5.04396718	**0.00027915*** **
GL;NM - PL;CM	0.61331436	0.16196746	43	3.78665168	**0.012589***
GL;NM - PL;NM	0.55054547	0.1448681	43	3.80032227	**0.01211735***
GL;NM - SL;OM	-0.24090719	0.1448681	43	-1.66294159	0.76469568
GL;NM - SL;CM	-0.07133593	0.1448681	43	-0.49241988	0.99988985
GL;NM - SL;NM	-0.5148051	0.1448681	43	-3.55361256	**0.02377215***
PL;OM - PL;CM	-0.11739559	0.16196746	43	-0.72480971	0.99813133
PL;OM - PL;NM	-0.18016448	0.1448681	43	-1.24364491	0.94165449
PL;OM - SL;OM	-0.97161714	0.1448681	43	-6.70690877	**1.17E-06*** **
PL;OM - SL;CM	-0.80204588	0.1448681	43	-5.53638706	**5.66E-05*** **
PL;OM - SL;NM	-1.24551505	0.1448681	43	-8.59757974	**2.42E-09*** **
PL;CM - PL;NM	-0.06276889	0.16196746	43	-0.38754011	0.99998231

$P_L;C_M$ - $S_L;O_M$	-0.85422155	0.16196746	43	-5.27403185	**0.000133***
$P_L;C_M$ - $S_L;C_M$	-0.68465029	0.16196746	43	-4.22708541	**0.00352483**
$P_L;C_M$ - $S_L;N_M$	-1.12811946	0.16196746	43	-6.96509938	**4.98E-07***
$P_L;N_M$ - $S_L;O_M$	-0.79145266	0.1448681	43	-5.46326385	**7.18E-05***
$P_L;N_M$ - $S_L;C_M$	-0.6218814	0.1448681	43	-4.29274215	**0.002896**
$P_L;N_M$ - $S_L;N_M$	-1.06535057	0.1448681	43	-7.35393482	**1.38E-07***
$S_L;O_M$ - $S_L;C_M$	0.16957126	0.1448681	43	1.17052171	0.95846879
$S_L;O_M$ - $S_L;N_M$	-0.27389791	0.1448681	43	-1.89067097	0.62348542
$S_L;C_M$ - $S_L;N_M$	-0.44346917	0.1448681	43	-3.06119268	0.08146444

*/**/***: Asterisks denote significant p-values (p: ***<0.001<**<0.01<*<0.05)

Table S2.C Tukey pairwise comparison results of the GLM model for the location x management interaction effect on the field capacity variable for the topsoil horizon (0-5 cm).

Contrast	Estimate	SE	df	t ratio	p value
GL;OM - GL;CM	-0.04940624	0.09983905	43	-0.49485883	0.99988567
GL;OM - GL;NM	0.31583523	0.09983905	43	3.16344385	0.06398162
GL;OM - PL;OM	-0.01520947	0.09983905	43	-0.15233987	0.99999999
GL;OM - PL;CM	-0.05857358	0.11162345	43	-0.52474262	0.99982239
GL;OM - PL;NM	-0.38171341	0.09983905	43	-3.82328768	**0.01136209***
GL;OM - SL;OM	-0.10068152	0.09983905	43	-1.00843825	0.98301584
GL;OM - SL;CM	-0.00162862	0.09983905	43	-0.01631243	1
GL;OM - SL;NM	-0.18718647	0.09983905	43	-1.87488234	0.63376956
GL;CM - GL;NM	0.36524146	0.09983905	43	3.65830267	**0.01793244***
GL;CM - PL;OM	0.03419677	0.09983905	43	0.34251895	0.9999932
GL;CM - PL;CM	-0.00916735	0.11162345	43	-0.08212743	1
GL;CM - PL;NM	-0.33230718	0.09983905	43	-3.32842886	**0.04262403***
GL;CM - SL;OM	-0.05127528	0.09983905	43	-0.51357942	0.99984883
GL;CM - SL;CM	0.04777762	0.09983905	43	0.47854639	0.99991125
GL;CM - SL;NM	-0.13778024	0.09983905	43	-1.38002352	0.89916679
GL;NM - PL;OM	-0.3310447	0.09983905	43	-3.31578372	**0.04400173***
GL;NM - PL;CM	-0.37440881	0.11162345	43	-3.35421281	**0.03993361***
GL;NM - PL;NM	-0.69754864	0.09983905	43	-6.98673153	**4.64E-07*****
GL;NM - SL;OM	-0.41651675	0.09983905	43	-4.17188209	**0.00415296****
GL;NM - SL;CM	-0.31746385	0.09983905	43	-3.17975628	0.06151716
GL;NM - SL;NM	-0.5030217	0.09983905	43	-5.03832619	**0.00028424*****
PL;OM - PL;CM	-0.04336411	0.11162345	43	-0.3884857	0.99998198
PL;OM - PL;NM	-0.36650394	0.09983905	43	-3.67094781	**0.01732491***
PL;OM - SL;OM	-0.08547205	0.09983905	43	-0.85609837	0.99412632
PL;OM - SL;CM	0.01358085	0.09983905	43	0.13602744	1
PL;OM - SL;NM	-0.171977	0.09983905	43	-1.72254247	0.72969517
PL;CM - PL;NM	-0.32313983	0.11162345	43	-2.89490985	0.11849802

$P_L;C_M$ - $S_L;O_M$	-0.04210793	0.11162345	43	-0.37723197	0.99998564
$P_L;C_M$ - $S_L;C_M$	0.05694496	0.11162345	43	0.51015233	0.99985624
$P_L;C_M$ - $S_L;N_M$	-0.12861289	0.11162345	43	-1.15220313	0.96207618
$P_L;N_M$ - $S_L;O_M$	0.28103189	0.09983905	43	2.81484944	0.14071733
$P_L;N_M$ - $S_L;C_M$	0.38008479	0.09983905	43	3.80697525	**0.01189385***
$P_L;N_M$ - $S_L;N_M$	0.19452694	0.09983905	43	1.94840534	0.58563899
$S_L;O_M$ - $S_L;C_M$	0.0990529	0.09983905	43	0.99212581	0.98466778
$S_L;O_M$ - $S_L;N_M$	-0.08650496	0.09983905	43	-0.8664441	0.99363532
$S_L;C_M$ - $S_L;N_M$	-0.18555785	0.09983905	43	-1.85856991	0.64435019

*/**/***: Asterisks denote significant p-values (p: ***<0.001<**<0.01<*<0.05)

Table S2.D Tukey pairwise comparison results of the GLM model for the location x management interaction effect on the bulk density variable for the topsoil horizon (0-5 cm).

Contrast	Estimate	SE	df	t ratio	p value
$G_L;O_M - G_L;C_M$	-0.21207879	0.06673976	43	-3.17769783	0.06182352
$G_L;O_M - G_L;N_M$	-0.15719812	0.06673976	43	-2.35538938	0.33373247
$G_L;O_M - P_L;O_M$	-0.15250475	0.06673976	43	-2.28506594	0.37319631
$G_L;O_M - P_L;C_M$	-0.07528302	0.07461732	43	-1.00892157	0.98296492
$G_L;O_M - P_L;N_M$	0.13176126	0.06673976	43	1.97425438	0.56863961
$G_L;O_M - S_L;O_M$	-0.21081995	0.06673976	43	-3.15883601	0.06469313
$G_L;O_M - S_L;C_M$	-0.242558	0.06673976	43	-3.63438532	**0.01913579***
$G_L;O_M - S_L;N_M$	-0.08056489	0.06673976	43	-1.2071499	0.95054079
$G_L;C_M - G_L;N_M$	0.05488067	0.06673976	43	0.82230845	0.99552339
$G_L;C_M - P_L;O_M$	0.05957404	0.06673976	43	0.89263189	0.99224575
$G_L;C_M - P_L;C_M$	0.13679576	0.07461732	43	1.83329777	0.66063317
$G_L;C_M - P_L;N_M$	0.34384005	0.06673976	43	5.15195221	**0.00019733*****
$G_L;C_M - S_L;O_M$	0.00125883	0.06673976	43	0.01886182	1
$G_L;C_M - S_L;C_M$	-0.03047921	0.06673976	43	-0.45668749	0.99993776
$G_L;C_M - S_L;N_M$	0.13151389	0.06673976	43	1.97054793	0.57107716
$G_L;N_M - P_L;O_M$	0.00469337	0.06673976	43	0.07032344	1
$G_L;N_M - P_L;C_M$	0.0819151	0.07461732	43	1.09780273	0.97146344
$G_L;N_M - P_L;N_M$	0.28895938	0.06673976	43	4.32964376	**0.00259145****
$G_L;N_M - S_L;O_M$	-0.05362183	0.06673976	43	-0.80344663	0.99617832
$G_L;N_M - S_L;C_M$	-0.08535988	0.06673976	43	-1.27899594	0.93206989
$G_L;N_M - S_L;N_M$	0.07663323	0.06673976	43	1.14823948	0.96282626
$P_L;O_M - P_L;C_M$	0.07722173	0.07461732	43	1.03490354	0.98005231
$P_L;O_M - P_L;N_M$	0.28426601	0.06673976	43	4.25932033	**0.00320128****
$P_L;O_M - S_L;O_M$	-0.0583152	0.06673976	43	-0.87377007	0.9932683
$P_L;O_M - S_L;C_M$	-0.09005325	0.06673976	43	-1.34931938	0.91004306
$P_L;O_M - S_L;N_M$	0.07193986	0.06673976	43	1.07791604	0.97442774
$P_L;C_M - P_L;N_M$	0.20704428	0.07461732	43	2.77474838	0.15302859

$P_L;C_M - S_L;O_M$	-0.13553693	0.07461732	43	-1.81642725	0.67141573
$P_L;C_M - S_L;C_M$	-0.16727498	0.07461732	43	-2.24177148	0.39860902
$P_L;C_M - S_L;N_M$	-0.00528187	0.07461732	43	-0.07078612	1
$P_L;N_M - S_L;O_M$	-0.34258121	0.06673976	43	-5.1330904	**0.00020968*** **
$P_L;N_M - S_L;C_M$	-0.37431926	0.06673976	43	-5.6086397	**4.46E-05*** **
$P_L;N_M - S_L;N_M$	-0.21232615	0.06673976	43	-3.18140428	0.06127285
$S_L;O_M - S_L;C_M$	-0.03173805	0.06673976	43	-0.47554931	0.99991537
$S_L;O_M - S_L;N_M$	0.13025506	0.06673976	43	1.95168611	0.58348188
$S_L;C_M - S_L;N_M$	0.16199311	0.06673976	43	2.42723542	0.29595871

*/**/***: Asterisks denote significant p-values (p: ***<0.001<**<0.01<*<0.05)

Table S2.E Tukey pairwise comparison results of the GLM model for the location x management interaction effect on the TOM variable for the topsoil horizon (0-5 cm).

Contrast	Estimate	SE	df	t ratio	p value
GL;OM - GL;CM	0.34591935	0.08189814	43	4.22377528	**0.00355978****
GL;OM - GL;NM	0.60503151	0.08189814	43	7.3876096	**1.23E-07*****
GL;OM - PL;OM	0.75736179	0.08189814	43	9.24760644	**3.11E-10*****
GL;OM - PL;CM	0.44853267	0.09156491	43	4.89852148	**0.00044387*****
GL;OM - PL;NM	0.32619228	0.08189814	43	3.98290201	**0.00721415****
GL;OM - SL;OM	0.64702098	0.08189814	43	7.90031324	**2.30E-08*****
GL;OM - SL;CM	0.82597389	0.08189814	43	10.0853799	**2.48E-11*****
GL;OM - SL;NM	0.09231552	0.08189814	43	1.12719913	0.96663088
GL;CM - GL;NM	0.25911216	0.08189814	43	3.16383432	0.06392164
GL;CM - PL;OM	0.41144244	0.08189814	43	5.02383116	**0.00029774*****
GL;CM - PL;CM	0.10261331	0.09156491	43	1.12066202	0.96775338
GL;CM - PL;NM	-0.01972707	0.08189814	43	-0.24087327	0.99999957
GL;CM - SL;OM	0.30110163	0.08189814	43	3.67653796	**0.01706246***
GL;CM - SL;CM	0.48005454	0.08189814	43	5.86160467	**1.94E-05*****
GL;CM - SL;NM	-0.25360384	0.08189814	43	-3.09657615	0.07500045
GL;NM - PL;OM	0.15233029	0.08189814	43	1.85999684	0.64342667
GL;NM - PL;CM	-0.15649884	0.09156491	43	-1.70915742	0.73771028
GL;NM - PL;NM	-0.27883923	0.08189814	43	-3.4047076	**0.03510175***
GL;NM - SL;OM	0.04198948	0.08189814	43	0.51270363	0.99985075
GL;NM - SL;CM	0.22094238	0.08189814	43	2.69777034	0.17899179
GL;NM - SL;NM	-0.51271599	0.08189814	43	-6.26041048	**5.18E-06*****
PL;OM - PL;CM	-0.30882913	0.09156491	43	-3.37278917	**0.03809064***
PL;OM - PL;NM	-0.43116952	0.08189814	43	-5.26470443	**0.00013708*****
PL;OM - SL;OM	-0.11034081	0.08189814	43	-1.3472932	0.91073373
PL;OM - SL;CM	0.06861209	0.08189814	43	0.8377735	0.99492156
PL;OM - SL;NM	-0.66504628	0.08189814	43	-8.12040731	**1.12E-08*****
PL;CM - PL;NM	-0.12234039	0.09156491	43	-1.33610563	0.91448712

$P_L;C_M$ - $S_L;O_M$	0.19848832	0.09156491	43	2.1677335	0.44378334
$P_L;C_M$ - $S_L;C_M$	0.37744122	0.09156491	43	4.12211657	**0.00480979****
$P_L;C_M$ - $S_L;N_M$	-0.35621715	0.09156491	43	-3.89032393	**0.00940232****
$P_L;N_M$ - $S_L;O_M$	0.3208287	0.08189814	43	3.91741123	**0.0087046****
$P_L;N_M$ - $S_L;C_M$	0.49978161	0.08189814	43	6.10247794	**8.74E-06*****
$P_L;N_M$ - $S_L;N_M$	-0.23387676	0.08189814	43	-2.85570288	0.12899591
$S_L;O_M$ - $S_L;C_M$	0.17895291	0.08189814	43	2.18506671	0.43303128
$S_L;O_M$ - $S_L;N_M$	-0.55470547	0.08189814	43	-6.77311411	**9.43E-07*****
$S_L;C_M$ - $S_L;N_M$	-0.73365837	0.08189814	43	-8.95818082	**7.70E-10*****

*/**/***: Asterisks denote significant p-values (p: ***<0.001<**<0.01<*<0.05)

Table S2.F Tukey pairwise comparison results of the GLM model for the location x management interaction effect on the normalized soil respiration variable for the topsoil horizon (0-5 cm).

Contrast	Estimate	SE	df	t ratio	p value
$G_L;O_M - G_L;C_M$	0.46576206	0.1497641	43	3.10997143	0.07267077
$G_L;O_M - G_L;N_M$	0.47120844	0.1497641	43	3.14633783	0.06665773
$G_L;O_M - P_L;O_M$	0.23953488	0.1497641	43	1.59941458	0.79981131
$G_L;O_M - P_L;C_M$	0.11333912	0.16744135	43	0.67688847	0.9988505
$G_L;O_M - P_L;N_M$	-0.11018622	0.1497641	43	-0.73573185	0.99792412
$G_L;O_M - S_L;O_M$	-0.36976267	0.1497641	43	-2.46896739	0.27527388
$G_L;O_M - S_L;C_M$	-0.26063893	0.1497641	43	-1.74032985	0.71891761
$G_L;O_M - S_L;N_M$	0.06722791	0.1497641	43	0.44889203	0.9999454
$G_L;C_M - G_L;N_M$	0.00544638	0.1497641	43	0.03636639	1
$G_L;C_M - P_L;O_M$	-0.22622718	0.1497641	43	-1.51055686	0.84444866
$G_L;C_M - P_L;C_M$	-0.35242294	0.16744135	43	-2.10475454	0.48361203
$G_L;C_M - P_L;N_M$	-0.57594828	0.1497641	43	-3.84570328	**0.01066765***
$G_L;C_M - S_L;O_M$	-0.83552473	0.1497641	43	-5.57893882	**4.92E-05*****
$G_L;C_M - S_L;C_M$	-0.72640099	0.1497641	43	-4.85030128	**0.00051714*****
$G_L;C_M - S_L;N_M$	-0.39853415	0.1497641	43	-2.6610794	0.19248115
$G_L;N_M - P_L;O_M$	-0.23167356	0.1497641	43	-1.54692325	0.8268577
$G_L;N_M - P_L;C_M$	-0.35786932	0.16744135	43	-2.13728163	0.46290289
$G_L;N_M - P_L;N_M$	-0.58139466	0.1497641	43	-3.88206968	**0.00962519****
$G_L;N_M - S_L;O_M$	-0.84097111	0.1497641	43	-5.61530522	**4.37E-05*****
$G_L;N_M - S_L;C_M$	-0.73184737	0.1497641	43	-4.88666767	**0.00046088*****
$G_L;N_M - S_L;N_M$	-0.40398053	0.1497641	43	-2.69744579	0.17910791
$P_L;O_M - P_L;C_M$	-0.12619576	0.16744135	43	-0.75367141	0.99754312
$P_L;O_M - P_L;N_M$	-0.34972109	0.1497641	43	-2.33514643	0.34484962
$P_L;O_M - S_L;O_M$	-0.60929755	0.1497641	43	-4.06838196	**0.00562988****
$P_L;O_M - S_L;C_M$	-0.50017381	0.1497641	43	-3.33974442	**0.04142397***
$P_L;O_M - S_L;N_M$	-0.17230697	0.1497641	43	-1.15052254	0.96239552
$P_L;C_M - P_L;N_M$	-0.22352534	0.16744135	43	-1.33494705	0.91487

$P_L;C_M - S_L;O_M$	-0.48310179	0.16744135	43	-2.88520004	0.12103091
$P_L;C_M - S_L;C_M$	-0.37397805	0.16744135	43	-2.23348681	0.40356124
$P_L;C_M - S_L;N_M$	-0.04611121	0.16744135	43	-0.27538724	0.99999877
$P_L;N_M - S_L;O_M$	-0.25957645	0.1497641	43	-1.73323554	0.72323286
$P_L;N_M - S_L;C_M$	-0.15045271	0.1497641	43	-1.004598	0.98341639
$P_L;N_M - S_L;N_M$	0.17741413	0.1497641	43	1.18462388	0.95553105
$S_L;O_M - S_L;C_M$	0.10912374	0.1497641	43	0.72863754	0.99806074
$S_L;O_M - S_L;N_M$	0.43699058	0.1497641	43	2.91785942	0.11268294
$S_L;C_M - S_L;N_M$	0.32786684	0.1497641	43	2.18922188	0.43046884

*/**/***: Asterisks denote significant p-values (p: ***<0.001<**<0.01<*<0.05)

Table S2.G Tukey pairwise comparison results of the GLM model for the location x management interaction effect on the C-CO2 efflux variable for the topsoil horizon (0-5 cm).

Contrast	Estimate	SE	df	t ratio	p value
GL;OM - GL;CM	0.85659043	0.17537883	43	4.88422947	**0.00046446*** **
GL;OM - GL;NM	1.1165591	0.17537883	43	6.36655594	**3.64E-06*** **
GL;OM - PL;OM	1.04429453	0.17537883	43	5.9545075	**1.43E-05*** **
GL;OM - PL;CM	0.55338038	0.19607949	43	2.82222476	0.13854081
GL;OM - PL;NM	0.2650882	0.17537883	43	1.51151772	0.84399648
GL;OM - SL;OM	0.30391921	0.17537883	43	1.73292988	0.72341829
GL;OM - SL;CM	0.60105607	0.17537883	43	3.42718723	**0.03312545***
GL;OM - SL;NM	0.20691212	0.17537883	43	1.17980105	0.95655168
GL;CM - GL;NM	0.25996868	0.17537883	43	1.48232647	0.85742077
GL;CM - PL;OM	0.1877041	0.17537883	43	1.07027803	0.97550345
GL;CM - PL;CM	-0.30321004	0.19607949	43	-1.54636288	0.82713607
GL;CM - PL;NM	-0.59150222	0.17537883	43	-3.37271175	**0.03809816***
GL;CM - SL;OM	-0.55267122	0.17537883	43	-3.15129959	0.06587168
GL;CM - SL;CM	-0.25553436	0.17537883	43	-1.45704224	0.86851559
GL;CM - SL;NM	-0.6496783	0.17537883	43	-3.70442842	**0.01580727***
GL;NM - PL;OM	-0.07226457	0.17537883	43	-0.41204844	0.99997164
GL;NM - PL;CM	-0.56317872	0.19607949	43	-2.87219599	0.1244918
GL;NM - PL;NM	-0.8514709	0.17537883	43	-4.85503822	**0.00050945*** **
GL;NM - SL;OM	-0.8126399	0.17537883	43	-4.63362606	**0.00102056****
GL;NM - SL;CM	-0.51550303	0.17537883	43	-2.93936871	0.10744735
GL;NM - SL;NM	-0.90964698	0.17537883	43	-5.18675489	**0.00017638*** **
PL;OM - PL;CM	-0.49091415	0.19607949	43	-2.50364866	0.25880854
PL;OM - PL;NM	-0.77920633	0.17537883	43	-4.44298978	**0.00183689****
PL;OM - SL;OM	-0.74037532	0.17537883	43	-4.22157762	**0.00358317****
PL;OM - SL;CM	-0.44323846	0.17537883	43	-2.52732027	0.24795279
PL;OM - SL;NM	-0.83738241	0.17537883	43	-4.77470645	**0.00065641*** **
PL;CM - PL;NM	-0.28829218	0.19607949	43	-1.47028221	0.86276829

P_L;C_M - S_L;O_M	-0.24946118	0.19607949	43	-1.27224516	0.9339759
P_L;C_M - S_L;C_M	0.04767569	0.19607949	43	0.24314469	0.99999954
P_L;C_M - S_L;N_M	-0.34646826	0.19607949	43	-1.76697862	0.70252174
P_L;N_M - S_L;O_M	0.038831	0.17537883	43	0.22141216	0.99999978
P_L;N_M - S_L;C_M	0.33596787	0.17537883	43	1.91566951	0.60713393
P_L;N_M - S_L;N_M	-0.05817608	0.17537883	43	-0.33171667	0.9999947
S_L;O_M - S_L;C_M	0.29713686	0.17537883	43	1.69425735	0.74653093
S_L;O_M - S_L;N_M	-0.09700709	0.17537883	43	-0.55312883	0.99973691
S_L;C_M - S_L;N_M	-0.39414395	0.17537883	43	-2.24738618	0.39526862

*/**/***: Asterisks denote significant p-values (p: ***<0.001<**<0.01<*<0.05)

Table S3. Identification table of the main cuticular hydrocarbons of *Lasius paralienus* (peaks retained for analyses in the present study) with peak compound classes and retention time..

Peak number	Retention time	Peak compound class	Peak identified compounds
1	18.674	Alkane	*n*-C28
2	20.233	Alkane	*n*-C29
3	20.679	Methylated Alkane	15-, 13-, 11-MeC29
4	21.332	Methylated Alkane	3-MeC29
5	21.431	Methylated Alkane	5,15-, 5,13-, 5,9-diMeC29
6	21.734	Alkane	*n*-C30
7	21.879	Methylated Alkane	5,9,11-triMeC29
8	22.197	Methylated Alkane	15-, 14-, 13-, 12-, 11-MeC30+9,12-diMeC30
9	22.606	Methylated Alkane	4-MeC30+7,14-diMeC30
10	22.921	Alkene + Methylated Alkane	X-C31:1+5,15-diMeC30+6,12-diMeC30
11	23.056	Methylated Alkane	4,14-, 4,12-, 4,10-, 4,8-diMeC30
12	23.266	Alkane	*n*-C31
13	23.497	Methylated Alkane	4,8,14-triMeC30
14	23.710	Methylated Alkane	15-, 13-, 11-, 9-MeC31
15	24.006	Methylated Alkane	5-MeC31
16	24.177	Methylated Alkane	9,15-, 9,13-diMeC31
17	24.293	Methylated Alkane	7,23-, 7-19-diMeC31+3-MeC31
18	24.462	Methylated Alkane	5,15-, 5,13-, 5,9-diMeC31
19	24.619	Methylated Alkane	7,11,15-triMeC31
20	24.883	Methylated Alkane	5,9,15-triMeC31+ 5,7,15-, 5,7,13-, 5,7,11-triMeC31
21	25.187	Methylated Alkane	16-, 15-, 14-, 13-, 12-, 11-, 10-MeC32
22	25.262	Methylated Alkane	9-MeC32+8,16-, 8,14-, 8,12-diMeC32
23	25.372	Methylated Alkane	6-MeC32
24	25.575	Methylated Alkane	4-MeC32+8,12,15-triMeC32+8,16, 8,14-, 8,12-diMeC32
25	25.785	Methylated Alkane	6,16-, 6,14-, 6,12-, 6,10-diMeC32+5,15-, 5,13-diMeC32
26	25.988	Methylated Alkane	4,16-, 4,14-, 4,12, 4,10-, 4,8-diMeC32

27	26.158	Alkane + Methylated Alkane	*n*-C33+6,10,16-triMeC32
28	26.413	Methylated Alkane	4,8,16-triMeC32
29	26.631	Methylated Alkane	17-, 15-, 13-, 11-MeC33
30	26.771	Methylated Alkane	7-MeC33
31	27.044	Methylated Alkane	5-MeC33+13,21-diMeC33 +11,17-diMeC33 +9,17-diMeC33
32	27.220	Methylated Alkane	7,25-, 7,21-, 7,19-, 7,17-diMeC33
33	27.349	Methylated Alkane	5,17-, 5,15-, 5,13-, 5,9-diMeC33
34	27.502	Methylated Alkane	7,11,15-triMeC33
35	27.743	Methylated Alkane	5,9,15-, 5,7,15-triMeC33
36	28.016	Methylated Alkane	17-, 16-, 15-, 14-, 13-, 12-MeC34
37	28.123	Methylated Alkane	8,16-diMeC34
38	28.406	Methylated Alkane	8,12,16-triMeC34
39	28.583	Methylated Alkane	6,16-, 6,14-, 6,12-diMeC34
40	28.786	Methylated Alkane	4,16-, 4,14-, 4,12-diMeC34
41	29.381	Methylated Alkane	17-, 15-, 13-MeC35
42	29.778	Methylated Alkane	13,21-diMeC35
43	29.896	Methylated Alkane	7,27-, 7,25-, 7,23-, 7,21-, 7,19-, 7,17-diMeC35
44	30.032	Methylated Alkane	5,19-, 5,17-, 5,15-, 5,13, 5,9-diMeC35
45	30.387	Methylated Alkane	5,9,15-triMeC35
46	30.775	Methylated Alkane	8,18-diMeC36
47	31.041	Methylated Alkane	14,22-diMeC36+12,24-diMeC36
48	31.975	Methylated Alkane	19-, 17-, 15-, 13-MeC37
49	32.337	Methylated Alkane	13,23-diMeC37

Table S4. Anova and tukey pairwise comparison results of the GLM models for location and management effects on the abundance of *Lasius paralienus* CHC profile for different compound classes.

Compound class	Variable	Pairwise comparison	Estimate	df	Test statistic (χ^2)	Test statistic (t ratio)	p-value
Whole profile	Location	Global test		2	34.2573913		**3.64E-08*** **
		Piverone - Pozzol Groppo	-0.003 12111	20		2.68706 026	**0.03617758**P*
		Piverone - Serralunga	0.0022 5781	20		-1.4261 6482	0.3469186
		Pozzol Groppo - Serralunga	0.0053 7892	20		-4.5560 511	**0.0005396**P*
	Management	Global test		2	1.47306644		0.47877083
Alkane profile	Location	Global test		2	7.69430552		**0.02134041***
		Piverone - Pozzol Groppo	-0.041 23182	20		2.11169 561	0.11274487
		Piverone - Serralunga	-0.018 64876	20		0.89297 245	0.65083395
		Pozzol Groppo - Serralunga	0.0225 8306	20		-1.9583 6977	0.14868934
	Management	Global test		2	6.45894004		**0.03957847***
		Conventional - Natural	0.0310 2951	20		-1.2641 7008	0.43097246
		Conventional - Organic	-0.015 39966	20		1.18587 928	0.47491993
		Natural - Organic	-0.046 42917	20		2.05023 142	0.12616567

Methylated alkane profile		Global test		2	27.4189389		**1.11E-06*****
	Location	Piverone - Pozzol Groppo	-0.00339838	20		2.29198914	0.08017849
		Piverone - Serralunga	0.00309553	20		-1.4706538	0.32566946
		Pozzol Groppo - Serralunga	0.00649391	20		-4.01496774	**0.00187929**P*
	Management	Global test		2	1.93448791		0.38012925
Mono-methylated alkane profile		Global test		2	24.5247252		**4.73E-06*****
	Location	Piverone - Pozzol Groppo	-0.01353025	20		2.22099917	0.09186741
		Piverone - Serralunga	0.01251235	20		-1.50761001	0.30865855
		Pozzol Groppo - Serralunga	0.0260426	20		-4.07986699	**0.00161825** P*
	Management	Global test		2	1.30213458		0.5214889

Di-methylated alkane profile	Location	Global test		2	28.8955584		**5.31E-07*** **
		Piverone - Pozzol Groppo	-0.01010003	20		2.34581254	0.07220742
		Piverone - Serralunga	0.0088097	20		-1.43434667	0.3429484
		Pozzol Groppo - Serralunga	0.01890973	20		-4.02664975	**0.0018294** p*
	Management	Global test		2	1.46107363		0.48165036
Tri-methylated alkane profile	Location	Global test		2	22.6885999		**1.18E-05*** **
		Piverone - Pozzol Groppo	-0.01267715	20		1.9520037	0.15036515
		Piverone - Serralunga	0.01490971	20		-1.50913827	0.30796778
		Pozzol Groppo - Serralunga	0.02758686	20		-3.50035948	**0.0060983** p*
	Management	Global test			2.35563862		0.30794955

*/**/***: Asterisks denote significant p-values (p: ***<0.001<**<0.01<*<0.05)

p*: Denote significant p-values for pairwise comparison controlled by using Tukey procedure

Table S5. Anova and tukey pairwise comparison results of the GLM models for location and management effects on the relative proportion of *Lasius paralienus* CHC profile for different compound classes.

Compound class	Variable	Pairwise comparison	Estimate	df	Test statistic (χ^2)	Test statistic (t ratio)	p-value
Alkane profile	Location	Global test		2	8.75026222		**0.01258649***
		Piverone - Pozzol Groppo	-0.02054778	20		0.4036 4556	0.91448567
		Piverone - Serralunga	-0.084339	20		1.9250 2324	0.15763871
		Pozzol Groppo - Serralunga	-0.06379122	20		2.0753 4671	0.12052787
	Management	Global test		2	14.2359534		**0.0008104*****
		Conventional - Natural	0.21834428	20		-2.275 85303	0.08271444
		Conventional - Organic	-0.02158597	20		0.8713 1735	0.66406883
		Natural - Organic	-0.23993025	20		2.5427 3164	**0.04871986P***
Methylated alkane profile	Location	Global test		2	5.64007032		0.05960385
	Management	Global test		2	5.85232312		0.05360239

Mono-methylated alkane profile	Location	Global test		2	6.42313901		**0.04029332***
		Piverone - Pozzol Groppo	0.00918219	20		-2.41502342	0.06299455
		Piverone - Serralunga	0.00294594	20		-0.8496004	0.67730421
		Pozzol Groppo - Serralunga	-0.00623625	20		1.8740118	0.17216411
	Management	Global test		2	1.53331016		0.4645644
Di-methylated alkane profile	Location	Global test		2	5.24402124		0.07265663
	Management	Global test		2	2.15697963		0.34010877
Tri-methylated alkane profile	Location	Global test		2	8.97107968		**0.0112708***
		Piverone - Pozzol Groppo	-0.00090081	20		0.17401819	0.98345792
		Piverone - Serralunga	0.01276922	20		-2.27383278	0.08303684
		Pozzol Groppo - Serralunga	0.01367003	20		-2.74690857	**0.03191164**P*
	Management	Global test		2	3.80074987		0.14951255

*/**/***: Asterisks denote significant p-values (p: ***<0.001<**<0.01<*<0.05)

P*: Denote significant p-values for pairwise comparison controlled by using Tukey procedure

Table S6. Anova and tukey pairwise comparison results of the GLM models for location and management effects on individual CHC peaks of *Lasius paralienus* CHC profile.

Peak number	Variable	Pairwise comparison	Estimate	df	Test statistic (χ^2)	Test statistic (t ratio)	p-value
1	Location	Global test		2	4.005737461		0.1349476
	Management	Global test		2	8.76899749		0.01246914
2	Location	Global test		2	4.35460375		0.11334694
	Management	Global test		2	6.8079834		0.03324032
3	Location	Global test		2	17.0822745		**0.00019527***
		Piverone - Pozzol Groppo	-5.1946 8144	20		2.01564993	0.1342875
		Piverone - Serralunga	2.35008077	20		-0.70182939	0.7651752
		Pozzol Groppo - Serralunga	7.54476221	20		-3.19087438	**0.01220364**p*
	Management	Global test		2	0.57868255		0.74875663
4	Location	Global test		2	12.5425604		**0.00188981***
		Piverone - Pozzol Groppo	-2.4609 7725	20		1.75053042	0.21170497
		Piverone - Serralunga	0.49804519	20		-0.28273212	0.95698565
		Pozzol Groppo - Serralunga	2.95902244	20		-2.5693351	**0.04614438**p*
	Management	Global test		2	1.40366786		0.49567544

5	Location	Global test		2	9.2918023		**0.00960087***
		Piverone - Pozzol Groppo	-8.19985996	20		1.39374347	0.36292223
		Piverone - Serralunga	3.02186462	20		-0.39040682	0.91975142
		Pozzol Groppo - Serralunga	11.2217246	20		-2.11251917	0.11257358
	Management	Global test		2	1.08918461		0.58007823
6	Location	Global test		2	11.6487375		**0.00295467***
		Piverone - Pozzol Groppo	-0.54455925	20		2.1844716	0.09843927
		Piverone - Serralunga	-0.0482013	20		0.16998081	0.98420992
		Pozzol Groppo - Serralunga	0.49635796	20		-2.78437306	**0.02948444**[p*]
	Management	Global test		2	5.64020777		0.05959975
7	Location	Global test		2	35.8013894		**1.68E-08***
		Piverone - Pozzol Groppo	-0.72290732	20		2.3627834	0.06984386
		Piverone - Serralunga	0.97860164	20		-2.00871484	0.13596742
		Pozzol Groppo - Serralunga	1.70150896	20		-4.28364995	**0.0010113**[p*]
	Management	Global test		2	2.63985304		0.26715493

8	Location	Global test		2	36.5724429		**1.14E-08***
		Piverone - Pozzol Groppo	-0.7084 4107	20		1.93441296	0.15507575
		Piverone - Serralunga	2.04996698	20		-2.89428243	**0.02332277**^{P*}
		Pozzol Groppo - Serralunga	2.75840805	20		-4.36433561	**0.00083951**^{P*}
	Management	Global test			1.12887841		0.56867897
9	Location	Global test		2	4.87171803		0.08752253
	Management	Global test		2	0.98936115		0.60976565
10	Location	Global test		2	30.1235618		**2.88E-07***
		Piverone - Pozzol Groppo	-3.6046 5835	20		1.8549912	0.17784517
		Piverone - Serralunga	8.09161532	20		-1.9896458	0.14067633
		Pozzol Groppo - Serralunga	11.6962737	20		-3.2362495	**0.01103426**^{P*}
	Management	Global test		2	0.99947836		0.60668888
11	Location	Global test		2	26.1673193		**2.08E-06***
		Piverone - Pozzol Groppo	-2.122 46464	20		2.09540028	0.11617978
		Piverone - Serralunga	1.98610301	20		-1.30027954	0.41138325
		Pozzol Groppo - Serralunga	4.10856766	20		-3.463727	**0.00662506**^{P*}
	Management	Global test		2	1.10325363		0.57601198

12	Location	Global test		2	12.4268666		**0.00200235***
		Piverone - Pozzol Groppo	-0.0943 8577	20		2.68315741	**0.03647335**^{p*}
		Piverone - Serralunga	-0.0429 526	20		1.14192622	0.50037373
		Pozzol Groppo - Serralunga	0.05143317	20		-2.48331861	0.05495002
	Management	Global test		2	5.42442762		0.06638967
13	Location	Global test		2	6.47021505		**0.03935597***
		Piverone - Pozzol Groppo	-1.5070 0003	20		1.22257807	0.45408172
		Piverone - Serralunga	0.04417243	20		-0.0294948	0.9995205
		Pozzol Groppo - Serralunga	1.55117246	20		-1.716263	0.22381875
	Management	Global test		2	13.5133201		**0.00116311***
		Conventional - Natural	-0.2770 7802	20		0.89814493	0.64766881
		Conventional - Organic	1.68509958	20		-2.35519651	0.07089187
		Natural - Organic	1.9621776	20		-2.8257966	**0.02700195**^{p*}

14	Location	Global test		2	25.4231624		**3.02E-06***
		Piverone - Pozzol Groppo	-0.1049 3479	20		2.31272149	0.07702065
		Piverone - Serralunga	0.08860307	20		-1.456232	0.3324663
		Pozzol Groppo - Serralunga	0.19353786	20		-4.18004049	**0.00128443**P*
	Management	Global test		2	1.69463592		0.42856281
15	Location	Global test		2	1.80834445		0.40487689
	Management	Global test		2	1.32081367		0.51664111
16	Location	Global test		2	15.6079804		**0.0004081***
		Piverone - Pozzol Groppo	-0.3169942	20		1.8228947	0.18776544
		Piverone - Serralunga	0.25174548	20		-1.07597828	0.53942436
		Pozzol Groppo - Serralunga	0.56873968	20		-3.21948215	**0.01145323**P*
	Management	Global test		2	3.26991811		0.19496035
17	Location	Global test		2	25.1315996		**3.49E-06***
		Piverone - Pozzol Groppo	-0.1422 2517	20		1.84873267	0.17974648
		Piverone - Serralunga	0.26315529	20		-2.08145977	0.11918812
		Pozzol Groppo - Serralunga	0.40538046	20		-3.79866906	**0.00308957**P*
	Management	Global test		2	1.49011994		0.47470583

18	Location	Global test		2	22.2111408		**1.50E-05***
		Piverone - Pozzol Groppo	-0.08904232	20		2.33478612	0.07378083
		Piverone - Serralunga	0.02504402	20		-0.51794957	0.86352398
		Pozzol Groppo - Serralunga	0.11408634	20		-3.50805905	**0.0059929**[P*]
	Management	Global test		2	0.84884537		0.65414733
19	Location	Global test		2	18.9886272		**7.53E-05***
		Piverone - Pozzol Groppo	-0.19719263	20		1.75357809	0.21065203
		Piverone - Serralunga	0.24903317	20		-1.42486439	0.3475522
		Pozzol Groppo - Serralunga	0.4462258	20		-3.16600629	**0.01289416**[P*]
	Management	Global test		2	3.26640694		0.19530292
20	Location	Global test		2	21.1021619		**2.62E-05***
		Piverone - Pozzol Groppo	-0.04523103	20		1.96572235	0.14677283
		Piverone - Serralunga	0.04229134	20		-1.26349185	0.43134472
		Pozzol Groppo - Serralunga	0.08752236	20		-3.38486044	**0.00791388**[P*]
	Management	Global test		2	2.46394247		0.29171697

21	Location	Global test		2	19.4545466		**5.96E-05***
		Piverone - Pozzol Groppo	-0.26512457	20		1.90968202	0.1618996
		Piverone - Serralunga	0.25185185	20		-1.26443846	0.43082519
		Pozzol Groppo - Serralunga	0.51697642	20		-3.35787644	**0.00840842**[p*]
	Management	Global test		2	3.02694859		0.2201438
22	Location	Global test		2	19.4214826		**6.06E-05***
		Piverone - Pozzol Groppo	-0.37163391	20		1.8714708	0.17291462
		Piverone - Serralunga	0.23594457	20		-0.84001273	0.68313167
		Pozzol Groppo - Serralunga	0.60757848	20		-2.94440296	**0.02093525**[p*]
	Management	Global test		2	4.07784625		0.13016881
23	Location	Global test		2	48.117922		**3.56E-11***
		Piverone - Pozzol Groppo	-2.03646035	20		2.90539065	**0.02277245**[p*]
		Piverone - Serralunga	0.71503275	20		-0.76417384	0.72871394
		Pozzol Groppo - Serralunga	2.75149309	20		-4.31521991	**0.00094025**[p*]
	Management	Global test		2	2.05859411		0.357258

24	Location	Global test		2	21.3629449		**2.30E-05***
		Piverone - Pozzol Groppo	-0.2270 9289	20		2.09201509	0.11690434
		Piverone - Serralunga	0.12048078	20		-0.81847897	0.69617616
		Pozzol Groppo - Serralunga	0.34757367	20		-3.2947674	**0.00968478**[P*]
	Management	Global test		2	3.77087418		0.15176271
25	Location	Global test		2	25.6273496		**2.72E-06***
		Piverone - Pozzol Groppo	-0.4194 9369	20		2.0871539	0.1179515
		Piverone - Serralunga	0.38060001	20		-1.26537919	0.4303092
		Pozzol Groppo - Serralunga	0.8000937	20		-3.4416999	**0.00696287**[P*]
	Management	Global test		2	2.12719458		0.34521175
26	Location	Global test		2	25.3177107		**3.18E-06***
		Piverone - Pozzol Groppo	-0.6791 9948	20		2.16423146	0.10225235
		Piverone - Serralunga	0.38524313	20		-0.88267097	0.65713357
		Pozzol Groppo - Serralunga	1.06444261	20		-3.37183975	**0.00814888**[P*]
	Management	Global test		2	2.32143765		0.31326092

27	Location	Global test		2	41.1434135		**1.16E-09***
		Piverone - Pozzol Groppo	-0.47222204	20		2.98386186	**0.01922021**P*
		Piverone - Serralunga	0.1694081	20		-0.81887407	0.69593743
		Pozzol Groppo - Serralunga	0.64163014	20		-4.52805985	**0.00057554**P*
	Management	Global test		2	1.73458302		0.42008781
28	Location	Global test		2	14.0936649		**0.00087016***
		Piverone - Pozzol Groppo	-0.50135887	20		1.6107477	0.26433082
		Piverone - Serralunga	0.28429641	20		-0.65622627	0.79102919
		Pozzol Groppo - Serralunga	0.78565528	20		-2.50712731	0.05237149
	Management	Global test		2	5.02270572		0.08115837
29	Location	Global test		2	15.5002507		**0.00043069***
		Piverone - Pozzol Groppo	-0.03617523	20		1.71395334	0.22465347
		Piverone - Serralunga	0.03874888	20		-1.34946695	0.38546071
		Pozzol Groppo - Serralunga	0.0749241	20		-3.33328907	**0.00888503**P*
	Management	Global test		2	2.02688059		0.36296811

30	Location	Global test		2	35.1137577		**2.37E-08***
		Piverone - Pozzol Groppo	-0.1015 2029	20		2.2380503	0.08893216
		Piverone - Serralunga	0.17323017	20		-2.48047886	0.05526512
		Pozzol Groppo - Serralunga	0.27475045	20		-4.73567937	**0.00035699**[p*]
	Management	Global test		2	1.80197421		0.40616853
31	Location	Global test		2	18.3276085		**0.00010476***
		Piverone - Pozzol Groppo	-0.0327 0398	20		2.18264365	0.09877854
		Piverone - Serralunga	0.014793	20		-0.78059456	0.71893883
		Pozzol Groppo - Serralunga	0.04749698	20		-3.52050835	**0.00582623**[p*]
	Management	Global test		2	3.39094605		0.1835124
32	Location	Global test		2	22.4266599		**1.35E-05***
		Piverone - Pozzol Groppo	-0.0292 0748	20		1.4567064	0.33224131
		Piverone - Serralunga	0.08600215	20		-2.4403334	0.05989747
		Pozzol Groppo - Serralunga	0.11520964	20		-3.70086551	**0.00386463**[p*]
	Management	Global test		2	1.12861151		0.56875487

33	Location	Global test		2	23.6034902		**7.49E-06***
		Piverone - Pozzol Groppo	-0.0690874	20		2.53687109	**0.04930454**[p*]
		Piverone - Serralunga	0.00298457	20		-0.0908931	0.99545664
		Pozzol Groppo - Serralunga	0.07207197	20		-3.55355667	**0.00540544**[p*]
	Management	Global test		2	1.15221469		0.5620821
34	Location	Global test		2	27.6623169		**9.84E-07***
		Piverone - Pozzol Groppo	-0.1165 6169	20		2.08417838	0.11859635
		Piverone - Serralunga	0.16260198	20		-1.85483734	0.17789172
		Pozzol Groppo - Serralunga	0.27916367	20		-3.90320708	**0.00243042**[p*]
	Management	Global test		2	1.87358093		0.39188358
35	Location	Global test		2	20.9909197		**2.77E-05***
		Piverone - Pozzol Groppo	-0.0328 9545	20		1.79276253	0.19746602
		Piverone - Serralunga	0.04908936	20		-1.70371852	0.22838019
		Pozzol Groppo - Serralunga	0.08198481	20		-3.46280028	**0.00663894**[p*]
	Management	Global test		2	1.92678264		0.38159657

36	Location	Global test		2	20.523966		**3.49E-05***
		Piverone - Pozzol Groppo	-0.2663 8182	20		1.83983133	0.18247814
		Piverone - Serralunga	0.32865572	20		-1.47914339	0.32170983
		Pozzol Groppo - Serralunga	0.59503754	20		-3.33544182	**0.00884228**[p*]
	Management	Global test		2	2.01933034		0.36434095
37	Location	Global test		2	23.1630213		**9.34E-06***
		Piverone - Pozzol Groppo	-0.1200 8703	20		1.95012676	0.15086217
		Piverone - Serralunga	0.13773424	20		-1.44641852	0.33714163
		Pozzol Groppo - Serralunga	0.25782127	20		-3.39479319	**0.00773905**[p*]
	Management	Global test		2	2.19059438		0.3344402
38	Location	Global test		2	31.8203745		**1.23E-07***
		Piverone - Pozzol Groppo	-0.1242 3467	20		2.25324021	0.08638638
		Piverone - Serralunga	0.15751017	20		-1.81653672	0.18978084
		Pozzol Groppo - Serralunga	0.28174484	20		-4.02015888	**0.00185695**[p*]
	Management	Global test		2	2.0566672		0.35760237

39	Location	Global test		2	43.9250023		**2.90E-10***
		Piverone - Pozzol Groppo	-2.59951566	20		2.16098129	0.10287632
		Piverone - Serralunga	3.26240305	20		-1.52017577	0.30300891
		Pozzol Groppo - Serralunga	5.8619187	20		-3.28476913	**0.00990348**^{P*}
	Management	Global test		2	1.08156062		0.5822937
40	Location	Global test		2	25.7436292		**2.57E-06***
		Piverone - Pozzol Groppo	-1.65207143	20		1.87961883	0.17051715
		Piverone - Serralunga	2.31763012	20		-1.52638986	0.30024038
		Pozzol Groppo - Serralunga	3.96970155	20		-3.14663341	**0.01345793**^{P*}
	Management	Global test		2	1.5090058		0.47024431
41	Location	Global test		2	22.8733517		**1.08E-05***
		Piverone - Pozzol Groppo	-0.076747	20		1.69434166	0.23183448
		Piverone - Serralunga	0.15869136	20		-2.16004503	0.10305667
		Pozzol Groppo - Serralunga	0.23543837	20		-3.76573796	**0.00333169**^{P*}
	Management	Global test		2	1.02651042		0.59854402

42	Location	Global test		2	28.3464505		**6.99E-07***
		Piverone - Pozzol Groppo	-0.0456064	20		2.10326578	0.11451091
		Piverone - Serralunga	0.06681646	20		-2.02052356	0.13311725
		Pozzol Groppo - Serralunga	0.11242286	20		-4.16067763	**0.00134311**[p*]
	Management	Global test		2	2.28072926		0.31970243
43	Location	Global test		2	23.7707398		**6.89E-06***
		Piverone - Pozzol Groppo	-0.30954307	20		1.87103981	0.17304217
		Piverone - Serralunga	0.45305499	20		-1.64781859	0.24954108
		Pozzol Groppo - Serralunga	0.76259806	20		-3.3516919	**0.00852592**[p*]
	Management	Global test		2	0.72376332		0.69636477
44	Location	Global test		2	26.6498334		**1.63E-06***
		Piverone - Pozzol Groppo	-0.66158119	20		2.36408196	0.06966587
		Piverone - Serralunga	-0.10531834	20		0.32350438	0.9441033
		Pozzol Groppo - Serralunga	0.55626285	20		-3.20148938	**0.01191984**[p*]
	Management	Global test		2	1.05381865		0.59042697

45	Location	Global test		2	22.1979729		**1.51E-05***
		Piverone - Pozzol Groppo	-0.57480504	20		1.86776327	0.17401433
		Piverone - Serralunga	0.63523647	20		-1.31047756	0.4059349
		Pozzol Groppo - Serralunga	1.21004151	20		-3.13148383	**0.01391518**P*
	Management	Global test		2	0.80866243		0.66742303
46	Location	Global test		2	13.6479407		**0.00108739***
		Piverone - Pozzol Groppo	-3.13113616	20		1.20104178	0.46626206
		Piverone - Serralunga	8.83214998	20		-1.48546366	0.31878201
		Pozzol Groppo - Serralunga	11.9632861	20		-2.21207923	0.09343615
	Management	Global test		2	2.3107316		0.31494231
47	Location	Global test		2	18.9410205		**7.71E-05***
		Piverone - Pozzol Groppo	-0.7340193	20		1.52741923	0.29978339
		Piverone - Serralunga	1.58149001	20		-1.94928833	0.15108461
		Pozzol Groppo - Serralunga	2.31550931	20		-3.32485833	**0.00905441**P*
	Management	Global test		2	1.52279652		0.46701297

48	Location	Global test		2	9.6084454		**0.00819507***
		Piverone - Pozzol Groppo	-0.58289794	20		0.98858601	0.59231715
		Piverone - Serralunga	1.66940893	20		-1.50679074	0.30902928
		Pozzol Groppo - Serralunga	2.25230687	20		-2.28757713	0.08086501
	Management	Global test		2	1.17199837		0.55654949
49	Location	Global test		2	27.2096386		**1.23E-06***
		Piverone - Pozzol Groppo	-0.31244825	20		2.03270738	0.13022868
		Piverone - Serralunga	0.47126095	20		-1.93589066	0.15467549
		Pozzol Groppo - Serralunga	0.7837092	20		-3.90909066	**0.00239777**^{p*}
	Management	Global test		2	0.98662351		0.61060088

*: Asterisks denote significant p-values controlled for FDR by using the Benjamini–Hochberg procedure

^{p*}: Denote significant p-values for pairwise comparison controlled by using Tukey procedure

Table S7. Permanova results for location, management, and location x management interaction effects on *Lasius paralienus* CHC profile for different compound classes.

Type of compound tested	Variable	Pairwise Comparison	df	Test statistic (F)	p-value
Whole profile	Location	Global test	2	15.48765	**0.00002*** **
		Pozzol Groppo - Piverone	1	10.3762723	**0.00622** **
		Pozzol Groppo - Serralunga	1	17.515169	**0.00028** **
		Piverone - Serralunga	1	3.60079757	0.05283
	Management	Global test	2	3.749864	**0.02565***
		Organic vineyard - Conventional vineyard	1	0.525180785	0.54602
		Organic vineyard - Natural area	1	2.30220252	0.11839
		Conventional vineyard - Natural area	1	0.70058051	0.48004
	Location x Management	Global test	3	1.897778331	0.13039
Alkane profile	Location	Global test	2	7.238773	**0.00205** **
		Pozzol Groppo - Piverone	1	10.1549504	**0.00932** **
		Pozzol Groppo - Serralunga	1	5.06723912	**0.02279***
		Piverone - Serralunga	1	0.40154283	0.66342

Alkane profile		Global test	2	10.36698	**0.00032*** **
	Management	Organic vineyard - Conventional vineyard	1	1.87789626	0.16973
		Organic vineyard - Natural area	1	7.39786984	**0.00965** **
		Conventional vineyard - Natural area	1	2.84306527	0.09744
	Location x Management	Global test	3	1.025847	0.40963
Methylated alkane profile	Location	Global test	2	16.29158	**0.00003*** **
		Pozzol Groppo - Piverone	1	9.70780358	**0.0092** **
		Pozzol Groppo - Serralunga	1	18.7430708	**0.00037*** **
		Piverone - Serralunga	1	4.19997886	**0.04165***
	Management	Global test	2	3.431698	**0.03901***
		Organic vineyard - Conventional vineyard	1	0.46662555	0.56716
		Organic vineyard - Natural area	1	2.06210929	0.14972
		Conventional vineyard - Natural area	1	0.5879108	0.50247
	Location x Management	Global test	3	1.980198	0.12954

Mono-methylated alkane profile	Location	Global test	2	15.8301536	**2.00E-05*** **
		Pozzol Groppo - Piverone	1	10.2359195	**0.00767** **
		Pozzol Groppo - Serralunga	1	17.6485009	**0.00021*** **
		Piverone - Serralunga	1	4.33447804	**0.03116***
	Management	Global test	2	3.35813572	**0.03595***
		Organic vineyard - Conventional vineyard	1	0.44250784	0.62618
		Organic vineyard - Natural area	1	1.57976925	0.20626
		Conventional vineyard - Natural area	1	0.56203618	0.52615
	Location x Management	Global test	3	2.23353916	0.08691
Di-methylated alkane profile	Location	Global test	2	14.4216285	**8.00E-05*** **
		Pozzol Groppo - Piverone	1	9.12102747	**0.01125***
		Pozzol Groppo - Serralunga	1	17.2284315	**0.00061*** **
		Piverone - Serralunga	1	3.55666962	0.061
	Management	Global test	2	3.00787797	0.05637
	Location x Management	Global test	3	1.94997562	0.13323

Tri-methylated alkane profile	Location	Global test	2	14.9106149	**0.00012*** **
		Pozzol Groppo - Piverone	1	8.22443208	**0.01653***
		Pozzol Groppo - Serralunga	1	20.3424983	**0.00047*** **
		Piverone - Serralunga	1	5.30109744	**0.03352***
	Management	Global test	2	2.53858956	0.09365
	Location x Management	Global test	3	1.0640673	0.38723

*/**/***: Asterisks denote significant p-values (p: ***<0.001<**<0.01<*<0.05)

Table S8. Indicator species analysis results concerning the *Lasius paralienus* CHCs' associations within the location variable.

Peak number	Associated groups Piverone	Pozzol Groppo	Serralunga	Index	Test statistic	p value
1	0	1	0	2	0.4588726	0.09521
2	0	1	0	2	0.49801001	0.05992
3	0	1	0	2	0.65258899	**0.00403****
4	0	1	0	2	0.65534731	**0.00292****
5	0	1	0	2	0.47091065	**0.04949***
6	0	1	0	2	0.66939545	**0.00309****
7	0	1	0	2	0.70473972	**0.00174****
8	0	1	0	2	0.755717	**0.00041*****
9	0	1	0	2	0.34288119	0.28368
10	0	1	0	2	0.67394907	**0.00295****
11	0	1	0	2	0.63544568	**0.00643****
12	0	1	0	2	0.62682885	**0.00801****
13	0	1	0	2	0.63763979	**0.00633****
14	0	1	0	2	0.69897586	**0.00094*****
15	0	1	0	2	0.38969213	0.19262
16	0	1	0	2	0.69441535	**0.00148****
17	0	1	0	2	0.66982164	**0.00314****
18	0	1	0	2	0.67654646	**0.00219****
19	0	1	0	2	0.71162311	**0.00125****
20	0	1	0	2	0.70281821	**0.00162****
21	0	1	0	2	0.73067193	**0.00063****
22	0	1	0	2	0.79602198	**0.00023*****
23	0	1	0	2	0.86176386	**3.00E-05*****
24	0	1	0	2	0.78157133	**0.00016*****
25	0	1	0	2	0.76767021	**0.00039*****
26	0	1	0	2	0.76872788	**0.00049*****

27	0	1	0	2	0.79849437	**0.00025*** **
28	0	1	0	2	0.74513382	**0.00077*** **
29	1	1	0	4	0.61321327	**0.00909** **
30	0	1	0	2	0.73484159	**0.00071*** **
31	0	1	0	2	0.66119291	**0.00344** **
32	1	1	0	4	0.7016416	**0.00163** **
33	0	1	0	2	0.71549944	**0.00098*** **
34	0	1	0	2	0.72821259	**0.00084*** **
35	1	1	0	4	0.68472918	**0.00256** **
36	0	1	0	2	0.72153792	**0.0011** **
37	0	1	0	2	0.7252856	**0.00102** **
38	0	1	0	2	0.74104778	**0.00053*** **
39	0	1	0	2	0.810468	**0.00028*** **
40	0	1	0	2	0.68979423	**0.00256** **
41	1	1	0	4	0.70935363	**0.00128** **
42	1	1	0	4	0.6871523	**0.00224** **
43	0	1	0	2	0.69237081	**0.00241** **
44	0	1	0	2	0.75351736	**8.00E-04*** **
45	0	1	0	2	0.60401472	**0.01099* **
46	0	1	0	2	0.55371226	**0.01261* **
47	0	1	0	2	0.70030686	**0.00146** **
48	0	1	0	2	0.68648656	**0.00281** **
49	0	1	0	2	0.7479353	**0.00044*** **

*/**/***: Asterisks denote significant p-values (p: ***<0.001<**<0.01<*<0.05)

Table S9.A. SIMPER analysis results of Pozzol Groppo - Serralunga contrast for the *Lasius paralienus* CHC profile location variable (ordered by decreasing average contribution of CHC peak to the average dissimilarity between observations from the two groups).

Peak	Average	Standard deviation	ratio	Average C_L	Average R_L	cumsum	p-value
20	0.01178563	0.00569903	2.06800459	3.57656767	2.05615104	0.03246266	**0.00013*** **
35	0.01173353	0.00555533	2.11212201	3.6315483	2.11828064	0.06478184	**0.00017*** **
37	0.0114133	0.005234	2.18060789	2.73485107	1.24067596	0.09621896	**3.00E-05*** **
38	0.01083469	0.00496898	2.18046754	2.65226324	1.22306289	0.12606235	**2.00E-05*** **
42	0.01051792	0.00555273	1.89418966	3.23094055	1.86484005	0.15503321	**2.00E-04*** **
32	0.01042863	0.00550753	1.89352084	3.19595672	1.85922869	0.18375813	**0.00121** **
34	0.01028974	0.00474854	2.16692522	2.55420371	1.20993345	0.21210049	**3.00E-05*** **
22	0.01026135	0.00420615	2.43960365	2.06704278	0.72856723	0.24036464	**1.00E-05*** **
19	0.01015294	0.00494395	2.05361076	2.1795049	0.86894011	0.26833019	**8.00E-05*** **
24	0.01001285	0.00466853	2.14475193	2.33400489	1.03006484	0.29590986	**2.00E-05*** **
33	0.00974095	0.00583157	1.67038197	3.43332983	2.21013382	0.32274061	**0.00097*** **
43	0.00964994	0.00478382	2.01720374	1.87613156	0.6275194	0.34932067	**0.00011*** **
41	0.00952775	0.00474284	2.0088713	2.53386506	1.29386263	0.37556419	**0.00031*** **
44	0.00944895	0.00512608	1.84330938	1.99488805	0.78653383	0.40159066	**0.00034*** **
28	0.00943642	0.00446726	2.11234792	1.79416408	0.57922457	0.4275826	**4.00E-05*** **
18	0.00925055	0.00578551	1.59891682	3.03228468	1.85379611	0.45306259	**0.0034** **

17	0.00911127	0.00458455	1.98738482	2.1606053	0.96668588	0.47815892	**0.00014*****
25	0.00898553	0.0040386	2.2249133	1.81797461	0.62859001	0.50290891	**2.00E-05*****
31	0.00887328	0.00564862	1.57087708	3.61195055	2.49441028	0.52734974	**0.00662****
36	0.0087193	0.00408075	2.13668738	1.88074803	0.74240153	0.55136642	**3.00E-05*****
29	0.00868741	0.00530459	1.63771725	3.19795944	2.10119824	0.57529528	**0.00366****
30	0.00852225	0.00342589	2.48760333	2.35184769	1.22900382	0.59876921	**1.00E-05*****
21	0.0081737	0.00395556	2.06638094	1.8606691	0.8005688	0.62128309	**4.00E-05*****
45	0.00810876	0.00429038	1.88998426	1.44432482	0.46309982	0.64361808	**0.00013*****
26	0.00787161	0.00372268	2.11449848	1.56176607	0.51692061	0.66529987	**3.00E-05*****
49	0.0078564	0.00371309	2.11586472	1.65626437	0.62120392	0.68693975	**2.00E-05*****
39	0.00775611	0.00388188	1.9980271	1.20517771	0.13784331	0.7083034	**1.00E-05*****
14	0.00763608	0.00458314	1.66612267	2.39684951	1.40208429	0.72933644	**0.00022*****
2	0.00762622	0.00549832	1.38700959	2.69082357	1.97570439	0.75034232	0.41775
27	0.0074746	0.0035268	2.11937319	1.76536127	0.75683699	0.77093058	**2.00E-05*****
16	0.00672143	0.00398948	1.68478857	1.55304301	0.6965752	0.78944428	**0.00163****
13	0.00612128	0.00381257	1.60555233	1.04632709	0.30242855	0.80630491	**0.00076*****
23	0.00603473	0.00247411	2.43915696	1.06616574	0.25775176	0.82292715	**1.00E-05*****
15	0.00601008	0.00453597	1.32498035	1.85868262	1.39667564	0.83948148	0.8626

48	0.00583928	0.00231973	2.51723079	1.00256318	0.25234141	0.85556537	**4.00E-05***
7	0.00579428	0.00308691	1.87704716	1.15041228	0.36952838	0.8715253	**4.00E-05***
40	0.00564945	0.00302712	1.86627583	0.92019152	0.18391646	0.88708631	**2.00E-05***
8	0.00520888	0.00201562	2.58425278	0.94122797	0.24643112	0.90143379	**1.00E-05***
12	0.00503131	0.00359657	1.39892002	2.69175277	2.10529866	0.91529217	0.67547
47	0.00463056	0.00231684	1.99865555	0.877948	0.26439862	0.92804671	**4.00E-05***
6	0.00404945	0.00250708	1.61520548	1.16760448	0.68765676	0.93920063	**0.01578***
11	0.00404532	0.00250508	1.61484264	0.69301401	0.17626503	0.95034317	**0.00015***
10	0.00342196	0.00201552	1.69780069	0.53712511	0.07260809	0.9597687	**7.00E-05***
4	0.0033123	0.002243	1.47672679	0.62933344	0.20911993	0.9688922	**0.00084***
9	0.00306953	0.00178987	1.71494702	0.55170864	0.30620974	0.977347	**0.03876***
46	0.00302201	0.00246554	1.22570234	0.47980719	0.06770603	0.98567092	**0.00013***
1	0.00207053	0.00151881	1.36325976	0.38508464	0.20641192	0.99137406	0.05386
5	0.00158644	0.00149189	1.06337747	0.25390951	0.06535196	0.99574381	**0.00533**
3	0.00154522	0.00114191	1.35318287	0.27908805	0.08904552	1	**0.00097***

*/**/***: Asterisks denote significant p-values (p: ***<0.001<**<0.01<*<0.05)

Table S9.B. SIMPER analysis results of Pozzol Groppo - Piverone contrast for the *Lasius paralienus* CHC profile location variable (ordered by decreasing average contribution of CHC peak to the average dissimilarity between observations from the two groups).

Peak	Average	Standard deviation	ratio	Average		cumsum	p-value
				CL	PL		
44	0.00826842	0.00394285	2.09706568	1.99488805	0.7647691	0.03715577	0.06241
33	0.0071368	0.00369089	1.93362726	3.43332983	2.37094544	0.0692264	0.62207
18	0.006767	0.00387294	1.74725135	3.03228468	2.06857445	0.09963525	0.69546
22	0.00638098	0.00298195	2.13987223	2.06704278	1.08108059	0.12830946	0.65622
15	0.00632598	0.00432329	1.46323198	1.85868262	1.13490552	0.15673651	0.64709
24	0.00603698	0.00260871	2.31416296	2.33400489	1.40074997	0.18386485	0.80863
20	0.00599868	0.00324188	1.850372	3.57656767	2.6963944	0.21082108	0.98361
39	0.00591735	0.00333799	1.77273016	1.20517771	0.28776738	0.23741186	**0.04158***
28	0.00578821	0.00335671	1.72436803	1.79416408	0.90493163	0.26342233	0.73049
26	0.00578308	0.00302339	1.9127808	1.56176607	0.7021426	0.28940974	0.21765
43	0.00575868	0.00296043	1.94521586	1.87613156	1.113811	0.31528749	0.91754
45	0.00573822	0.00285781	2.00791019	1.44432482	0.80103188	0.34107331	0.51658
37	0.00573251	0.00314167	1.82466628	2.73485107	1.86376874	0.36683345	0.98383
2	0.0057325	0.00328923	1.74281037	2.69082357	1.97958206	0.39259356	0.89484
38	0.00563263	0.00292589	1.92509824	2.65226324	1.80947092	0.41790488	0.9617
12	0.00561275	0.00295158	1.90160748	2.69175277	1.89239672	0.44312686	0.32805

27	0.00560674	0.0029342	1.91082184	1.76536127	0.90817614	0.46832186	0.12594
25	0.00556017	0.0030218	1.84001589	1.81797461	0.97302643	0.49330757	0.70825
19	0.005435	0.00262627	2.06947291	2.1795049	1.41207232	0.51773082	0.96545
35	0.00526758	0.00278487	1.89150135	3.6315483	2.90326123	0.54140173	0.99622
34	0.00518521	0.0024406	2.12456357	2.55420371	1.80594647	0.56470248	0.9788
31	0.00489293	0.00247382	1.97788178	3.61195055	2.88790931	0.58668983	0.95377
23	0.00487817	0.00215201	2.26679603	1.06616574	0.32658692	0.60861083	**0.01231***
36	0.00475097	0.00247586	1.91891532	1.88074803	1.19110817	0.62996026	0.95574
42	0.00467477	0.0022704	2.05901029	3.23094055	2.55233094	0.65096725	0.98632
32	0.00454087	0.00262208	1.73178225	3.19595672	2.75188501	0.67137256	0.99827
17	0.00450152	0.00275619	1.63323837	2.1606053	1.54658124	0.69160102	0.98552
21	0.00442131	0.00244829	1.80588036	1.8606691	1.18272733	0.71146905	0.95039
13	0.00424096	0.00325866	1.30144065	1.04632709	0.40567091	0.73052662	0.54429
40	0.00421864	0.0021935	1.92324433	0.92019152	0.36552961	0.74948389	0.20432
14	0.0041088	0.00233842	1.75708346	2.39684951	1.77592442	0.76794761	0.90857
49	0.0039228	0.00210355	1.86485149	1.65626437	1.0509889	0.7855755	0.96329
7	0.00385561	0.00232554	1.65794291	1.15041228	0.62051029	0.80290142	0.58495
16	0.00385527	0.00203587	1.89367739	1.55304301	0.98541972	0.82022586	0.92457

41	0.0038522	0.00223564	1.72308695	2.53386506	1.99415868	0.83753649	0.99709
29	0.00384466	0.00240315	1.59984037	3.19795944	2.65250002	0.85481321	0.99163
30	0.00379261	0.00205619	1.8444856	2.35184769	1.77829584	0.87185603	0.99459
6	0.00351464	0.00210862	1.6667976	1.16760448	0.68374336	0.88764976	0.37648
11	0.00321267	0.0019981	1.60786281	0.69301401	0.28152814	0.90208654	0.19072
8	0.00271812	0.0015281	1.77875914	0.94122797	0.54994772	0.91430095	0.93212
10	0.00269226	0.00176173	1.52818734	0.53712511	0.17619718	0.92639913	0.13006
48	0.00264757	0.00152688	1.73397294	1.00256318	0.62355831	0.93829651	0.99905
4	0.00259908	0.00202486	1.2835797	0.62933344	0.24625329	0.94997597	0.34502
9	0.00252974	0.00138674	1.82422985	0.55170864	0.39133931	0.96134383	0.6573
47	0.00225295	0.00160882	1.40037134	0.877948	0.53185215	0.97146789	0.97883
46	0.00212079	0.00199576	1.0626504	0.47980719	0.20196654	0.98099809	0.50704
1	0.00158253	0.00111092	1.42451637	0.38508464	0.21625613	0.98810948	0.74644
5	0.00135205	0.00129075	1.04749495	0.25390951	0.08475216	0.99418519	0.37586
3	0.00129399	0.00094553	1.36853911	0.27908805	0.11290323	1	0.21948

*/**/***: Asterisks denote significant p-values (p: ***<0.001<**<0.01<*<0.05)

Table S9.C. SIMPER analysis results of Piverone - Serralunga contrast for the *Lasius paralienus* CHC profile location variable (ordered by decreasing average contribution of CHC peak to the average dissimilarity between observations from the two groups).

Peak	Average	Standard deviation	ratio	Average		cumsum	p-value
				P_L	R_L		
32	0.00977648	0.0060476	1.61658764	2.75188501	1.85922869	0.04126659	**0.03136***
35	0.00936404	0.00524136	1.78656536	2.90326123	2.11828064	0.08079226	0.11082
20	0.00840688	0.00492195	1.70803959	2.6963944	2.05615104	0.11627775	0.4752
42	0.00795367	0.00600886	1.32365748	2.55233094	1.86484005	0.14985026	0.36153
41	0.00792152	0.00507346	1.56136413	1.99415868	1.29386263	0.18328703	0.09599
15	0.00784554	0.00628438	1.24841985	1.13490552	1.39667564	0.21640311	0.20767
37	0.00782767	0.00444263	1.76194396	1.86376874	1.24067596	0.24944376	0.49583
29	0.0073984	0.00526458	1.40531529	2.65250002	2.10119824	0.28067243	0.256
34	0.0071056	0.00423851	1.67643748	1.80594647	1.20993345	0.31066522	0.4559
2	0.00709926	0.00490517	1.44730141	1.97958206	1.97570439	0.34063125	0.5742
38	0.00704179	0.00453587	1.552466	1.80947092	1.22306289	0.3703547	0.66514
19	0.00690553	0.00434026	1.5910413	1.41207232	0.86894011	0.39950297	0.62563
31	0.00684144	0.00499102	1.37074855	2.88790931	2.49441028	0.42838073	0.58512
17	0.00676453	0.00456658	1.48131077	1.54658124	0.96668588	0.45693385	0.3891
43	0.00627806	0.00411211	1.52672411	1.113811	0.6275194	0.48343357	0.79417
30	0.00614764	0.00329236	1.86724571	1.77829584	1.22900382	0.50938279	0.18038

18	0.00598555	0.00470011	1.27349081	2.06857445	1.85379611	0.53464782	0.89566
36	0.00574117	0.00328002	1.75034528	1.19110817	0.74240153	0.55888136	0.66599
33	0.00568314	0.00430552	1.31996555	2.37094544	2.21013382	0.58286993	0.96347
24	0.0056213	0.0035848	1.56809226	1.40074997	1.03006484	0.60659747	0.92856
12	0.00551807	0.00367527	1.50140535	1.89239672	2.10529866	0.62988928	0.34179
22	0.00533196	0.00292585	1.82236139	1.08108059	0.72856723	0.65239552	0.98067
21	0.00525168	0.00329869	1.59205324	1.18272733	0.8005688	0.67456291	0.74567
14	0.00510518	0.00466196	1.09507109	1.77592442	1.40208429	0.6961119	0.71242
28	0.005088	0.00262947	1.93498978	0.90493163	0.57922457	0.71758838	0.95321
49	0.00500173	0.00351816	1.42168798	1.0509889	0.62120392	0.7387007	0.67752
25	0.00485208	0.00269657	1.79935033	0.97302643	0.62859001	0.75918137	0.96409
16	0.00479632	0.00369634	1.29758438	0.98541972	0.6965752	0.77942666	0.6833
45	0.0046075	0.00228594	2.0155844	0.80103188	0.46309982	0.79887493	0.96654
48	0.00449985	0.00184102	2.44421848	0.62355831	0.25234141	0.81786884	0.08351
44	0.00442246	0.00306618	1.44233515	0.7647691	0.78653383	0.83653605	0.99987
26	0.00354544	0.00259724	1.36508062	0.7021426	0.51692061	0.85150137	0.99912
8	0.00325964	0.00214117	1.52236646	0.54994772	0.24643112	0.86526034	0.59931
47	0.0031882	0.00206405	1.54463044	0.53185215	0.26439862	0.87871775	0.53604

7	0.00314172	0.00203889	1.54089459	0.62051029	0.36952838	0.89197897	0.95339
13	0.00299236	0.00234762	1.27463532	0.40567091	0.30242855	0.90460974	0.96908
27	0.00280534	0.00195967	1.43153567	0.90817614	0.75683699	0.9164511	0.99981
6	0.00270417	0.00209294	1.29204012	0.68374336	0.68765676	0.92786541	0.93696
40	0.00238269	0.00157419	1.51359664	0.36552961	0.18391646	0.93792274	0.99806
9	0.00226864	0.00169111	1.34151267	0.39133931	0.30620974	0.94749868	0.90917
39	0.00195988	0.00127156	1.54131475	0.28776738	0.13784331	0.95577135	0.99967
11	0.00162934	0.00128067	1.2722525	0.28152814	0.17626503	0.96264879	0.997
46	0.00161204	0.00090944	1.7725631	0.20196654	0.06770603	0.96945321	0.85391
23	0.00152204	0.0011069	1.37504063	0.32658692	0.25775176	0.97587774	0.99998
1	0.00146853	0.00110536	1.32855742	0.21625613	0.20641192	0.9820764	0.87407
4	0.00146538	0.00139212	1.05262571	0.24625329	0.20911993	0.9882618	0.98746
10	0.00135135	0.00124171	1.08830214	0.17619718	0.07260809	0.99396586	0.99327
3	0.00073814	0.00061282	1.20449405	0.11290323	0.08904552	0.99708154	0.98176
5	0.00069141	0.0005261	1.31422254	0.08475216	0.06535196	1	0.98322

*/**/***: Asterisks denote significant p-values (p: ***<0.001<**<0.01<*<0.05)

Table S10. Indicator species analysis results concerning the *Lasius paralienus* CHCs′ associations within the management variable.

Peak number	Associated groups			Index	Test statistic	p value
	Conventional vineyard	Natural area	Organic vineyard			
1	0	0	1	3	0.55522782	**0.02716***
2	1	0	1	5	0.5170272	**0.04911***
3	1	0	1	5	0.12692793	0.85386
4	0	1	0	2	0.1719985	0.77217
5	1	1	0	4	0.15831659	0.85235
6	0	0	1	3	0.42967332	0.1354
7	1	1	0	4	0.23787295	0.56356
8	0	1	0	2	0.205119	0.64319
9	0	1	1	6	0.27472045	0.45657
10	1	1	0	4	0.15234288	0.80103
11	0	1	0	2	0.28466945	0.44972
12	1	0	1	5	0.51047377	0.05405
13	1	1	0	4	0.40748743	0.16402
14	0	1	0	2	0.27285455	0.46447
15	0	1	1	6	0.22652068	0.58535
16	0	1	0	2	0.4168676	0.15353
17	1	1	0	4	0.19242373	0.68271
18	0	1	0	2	0.26506131	0.48557
19	1	1	0	4	0.23658492	0.56049
20	1	1	0	4	0.25629107	0.50512
21	1	1	0	4	0.29119866	0.41248
22	1	1	0	4	0.317061	0.34892
23	1	1	0	4	0.20630399	0.64463
24	0	1	0	2	0.37781785	0.21873
25	1	1	0	4	0.26747684	0.47628
26	1	1	0	4	0.24159341	0.54746

27	1	1	0	4	0.21670638	0.61957
28	1	1	0	4	0.31669389	0.3524
29	0	1	0	2	0.29528704	0.40511
30	1	1	0	4	0.22801828	0.5806
31	0	1	0	2	0.42932268	0.1375
32	1	1	0	4	0.23969525	0.5495
33	0	1	0	2	0.3160727	0.35406
34	1	1	0	4	0.2246649	0.59034
35	1	1	0	4	0.23752528	0.55474
36	0	1	0	2	0.2744991	0.45431
37	1	1	0	4	0.25422104	0.51079
38	0	1	0	2	0.31187004	0.36133
39	1	1	0	4	0.18421876	0.70395
40	1	1	0	4	0.23181899	0.58081
41	0	1	0	2	0.2214284	0.60114
42	0	1	0	2	0.34477673	0.28672
43	1	1	0	4	0.22990678	0.57674
44	0	1	0	2	0.31321389	0.36056
45	1	1	0	4	0.25779982	0.50849
46	1	0	0	1	0.22966095	0.64793
47	0	1	0	2	0.32350501	0.33329
48	0	1	0	2	0.27747583	0.44292
49	0	1	0	2	0.31257507	0.35847

*/**/***: Asterisks denote significant p-values (p: ***<0.001<**<0.01<*<0.05)

Table S11.A. SIMPER analysis results of organic vineyard - conventional vineyard contrast for the *Lasius paralienus* CHC profile management variable (ordered by decreasing average contribution of CHC peak to the average dissimilarity between observations from the two groups).

Peak	Average	Standard deviation	ratio	Average		cumsum	p-value
				B_M	C_M		
32	0.0089181	0.00512872	1.73885343	2.30993756	2.6529814	0.03301494	0.06698
42	0.00843285	0.00547546	1.54011855	2.28759096	2.48049226	0.06423347	0.11411
20	0.00804156	0.00521545	1.54187347	2.47929996	2.84738794	0.09400345	0.68569
35	0.00800208	0.00536609	1.4912318	2.59292481	2.92644305	0.12362726	0.69328
18	0.00781588	0.00571109	1.36854487	2.20407451	2.22963451	0.15256176	0.29012
38	0.00780218	0.00496717	1.57074873	1.61689374	1.90558443	0.18144555	0.23518
31	0.00772799	0.00506133	1.5268708	2.74571454	2.94424	0.2100547	0.16971
15	0.00769574	0.00654717	1.1754291	1.66487034	1.24725661	0.23854444	0.15868
19	0.0076212	0.00407716	1.86924002	1.24466867	1.56731111	0.26675822	0.19642
29	0.0075602	0.00538157	1.40483105	2.43504979	2.61445529	0.2947462	0.11737
37	0.0075415	0.00503373	1.49819369	1.66672871	2.00320788	0.32266495	0.68558
33	0.00750887	0.00520785	1.4418372	2.54197391	2.57154836	0.3504629	0.50172
41	0.007336	0.00513884	1.42756053	1.71999051	1.93093442	0.37762087	0.1892
34	0.00733557	0.00446234	1.64388576	1.62926777	1.88282618	0.40477726	0.27174
43	0.00713226	0.00443756	1.60724931	0.99409978	1.23415195	0.431181	0.26998
24	0.00708609	0.00446196	1.58811334	1.30321831	1.62699754	0.45741381	0.30614

17	0.00708259	0.00392024	1.80667244	1.35843436	1.64121346	0.48363365	0.16201
44	0.00687842	0.00463364	1.48445246	1.0434618	1.15281126	0.50909764	0.54534
14	0.00671096	0.00461556	1.45398706	1.73779168	1.77561172	0.53394173	**0.04981***
2	0.00652748	0.00499452	1.30692908	2.57961473	2.15019135	0.55810657	0.8369
22	0.00644639	0.004199	1.53521843	1.01719671	1.38220621	0.58197118	0.68983
21	0.00621989	0.00387581	1.60479825	1.06528079	1.33713591	0.60499732	0.12957
25	0.00614901	0.00406087	1.5142097	0.92379606	1.2392122	0.62776106	0.29858
36	0.00613355	0.00396213	1.54804241	1.06435092	1.28048308	0.65046755	0.39489
28	0.00591162	0.00428203	1.38056445	0.82400911	1.18120343	0.67235245	0.75569
16	0.00587281	0.00357303	1.64364736	0.91418072	1.01521779	0.69409368	0.09099
45	0.0056443	0.00404719	1.39461991	0.69627002	0.97110207	0.71498895	0.60768
30	0.00562072	0.00370965	1.51516208	1.59141553	1.83217878	0.73579695	0.51868
49	0.00546047	0.00375998	1.45226085	0.95253391	1.05764208	0.75601171	0.36205
26	0.00531203	0.00376557	1.41068488	0.7624231	0.98652938	0.77567693	0.47217
27	0.00482851	0.0037152	1.29966238	0.9971846	1.30188995	0.79355215	0.53206
39	0.00468958	0.00412962	1.13559727	0.4182063	0.65209041	0.81091304	0.40075
12	0.00447285	0.00308988	1.44757902	2.45647601	2.30397143	0.82747158	0.89328
48	0.0039021	0.00264594	1.47474694	0.49052224	0.61346474	0.8419172	0.58715

7	0.00386943	0.00243602	1.58842548	0.5728204	0.75116682	0.85624188	0.61442
40	0.00379759	0.00296699	1.27994562	0.36071526	0.57446472	0.87030062	0.48659
13	0.00369549	0.00309469	1.19413694	0.3298577	0.69798633	0.88398136	0.89566
8	0.00369115	0.00214739	1.71890545	0.4880723	0.5638115	0.89764607	0.1083
23	0.0036458	0.00302627	1.20471773	0.45069321	0.64815939	0.91114286	0.39211
47	0.00342441	0.0022159	1.54538049	0.45184454	0.53021789	0.92382008	0.24767
6	0.00322321	0.00234041	1.37719458	1.00832908	0.7979346	0.93575243	0.67321
9	0.00286547	0.00205292	1.39580711	0.42402013	0.3076339	0.94636046	0.19865
11	0.00263682	0.00211392	1.24735883	0.32058976	0.34427822	0.95612202	0.72032
10	0.00249634	0.00214942	1.16139704	0.20487395	0.32992903	0.96536349	0.14864
46	0.0024201	0.00236142	1.02484995	0.17450502	0.35194845	0.97432272	0.14393
4	0.00229681	0.00181965	1.26222679	0.34882087	0.31998293	0.98282555	0.64348
1	0.00199183	0.00167916	1.18620591	0.37733501	0.19678679	0.99019935	0.12954
3	0.0013586	0.00102398	1.32679368	0.15835234	0.18199415	0.99522892	**0.03956***
5	0.00128878	0.0014354	0.89785084	0.10240592	0.15876532	1	0.3924

*/**/***: Asterisks denote significant p-values (p: ***<0.001<**<0.01<*<0.05)

Table S11.B. SIMPER analysis results of organic vineyard - natural area contrast for the *Lasius paralienus* CHC profile management variable (ordered by decreasing average contribution of CHC peak to the average dissimilarity between observations from the two groups).

Peak	Average	Standard deviation	ratio	Average		cumsum	p-value
				B_M	N_M		
2	0.01048503	0.00806058	1.30077849	2.57961473	1.55088826	0.04034466	**0.00479****
20	0.00904698	0.00538844	1.67896158	2.47929996	3.0904017	0.07515594	0.19993
35	0.00887531	0.00545681	1.6264656	2.59292481	3.14884496	0.10930666	0.23712
37	0.00820594	0.00524597	1.56423654	1.66672871	2.24958819	0.14088178	0.30227
19	0.00737707	0.00395594	1.86480851	1.24466867	1.70465376	0.16926754	0.37526
22	0.00721451	0.00457357	1.57743627	1.01719671	1.67256205	0.19702778	0.20529
33	0.00719312	0.00505798	1.42213278	2.54197391	3.09203049	0.22470571	0.61469
32	0.00711612	0.00443041	1.60619941	2.30993756	2.78783656	0.25208738	0.81981
38	0.00697157	0.00462857	1.50620338	1.61689374	2.27423347	0.27891282	0.70248
44	0.00694767	0.00450866	1.54095977	1.0434618	1.62010591	0.30564631	0.49017
28	0.00692596	0.00446701	1.55046787	0.82400911	1.46753847	0.33229626	0.15572
24	0.00688965	0.00452667	1.52201328	1.30321831	2.05041065	0.35880649	0.45007
31	0.00685855	0.00482719	1.42081487	2.74571454	3.49334275	0.38519707	0.57995
34	0.00681093	0.004282	1.59059604	1.62926777	2.09219356	0.4114044	0.61707
12	0.0067185	0.00500703	1.34181477	2.45647601	1.77999124	0.4372561	**0.02756***
42	0.00665962	0.00475744	1.39983468	2.28759096	2.94612665	0.46288124	0.80503

43	0.00658539	0.00413264	1.59350686	0.99409978	1.45582806	0.48822074	0.6514
17	0.00635797	0.00369911	1.71878457	1.35843436	1.65066705	0.51268519	0.59558
18	0.00627554	0.00445694	1.40803665	2.20407451	2.65736994	0.53683243	0.84939
41	0.0059603	0.00403865	1.47581386	1.71999051	2.14878074	0.55976669	0.85645
45	0.00594102	0.00375823	1.58080026	0.69627002	1.1468143	0.58262675	0.38189
15	0.00592022	0.00479322	1.23512495	1.66487034	1.49327501	0.60540679	0.79306
13	0.00579667	0.00396789	1.46089644	0.3298577	0.98178354	0.62771143	**0.01979***
36	0.00577433	0.00384947	1.50003415	1.06435092	1.55085973	0.64993011	0.65192
25	0.00570221	0.00370584	1.53871159	0.92379606	1.37171743	0.67187129	0.64662
29	0.00564628	0.00425169	1.32800693	2.43504979	2.95428467	0.69359723	0.87318
30	0.00558788	0.00365188	1.53013678	1.59141553	1.93309532	0.71509849	0.53725
21	0.00511782	0.00335132	1.52710896	1.06528079	1.54532747	0.73479103	0.81689
26	0.005089	0.00346157	1.47014399	0.7624231	1.17633759	0.75437268	0.63448
49	0.00502627	0.00351573	1.42965168	0.95253391	1.36714621	0.77371293	0.67053
16	0.00484258	0.00299021	1.61947888	0.91418072	1.43093644	0.79234637	0.66951
27	0.00482784	0.00290657	1.66101061	0.9971846	1.2299761	0.81092312	0.51712
14	0.00434725	0.00305151	1.42462324	1.73779168	2.1060541	0.82765064	0.91341
7	0.00431276	0.00268469	1.60642827	0.5728204	0.88838826	0.84424544	0.22921

39	0.00428398	0.00336778	1.27204949	0.4182063	0.6587579	0.8607295	0.6744
6	0.00415277	0.00310211	1.33869052	1.00832908	0.59179162	0.87670868	**0.04242***
48	0.00402598	0.00247818	1.62457198	0.49052224	0.79434265	0.89219998	0.41626
40	0.00367211	0.00237845	1.5439062	0.36071526	0.60784478	0.90632964	0.59093
23	0.00333584	0.00235063	1.4191225	0.45069321	0.64900743	0.9191654	0.67957
47	0.00306102	0.00193817	1.5793333	0.45184454	0.72900315	0.93094371	0.66234
8	0.00293906	0.00201737	1.45687832	0.4880723	0.68567954	0.94225274	0.87523
11	0.00290788	0.00221171	1.31476615	0.32058976	0.55559365	0.95344177	0.39796
4	0.00252926	0.00231933	1.09051247	0.34882087	0.45604464	0.96317396	0.36539
9	0.00228338	0.00146748	1.55598527	0.42402013	0.52296087	0.97196002	0.91196
1	0.00216376	0.00157623	1.37273906	0.37733501	0.13957687	0.9802858	0.07578
10	0.00176079	0.00129831	1.35621109	0.20487395	0.26819019	0.98706103	0.9225
46	0.00156432	0.00114522	1.36595863	0.17450502	0.2284312	0.99308026	0.89767
5	0.00103056	0.0007725	1.33405658	0.10240592	0.17414684	0.99704569	0.73703
3	0.00076778	0.00066478	1.15494768	0.15835234	0.13436817	1	0.97907

*/**/***: Asterisks denote significant p-values (p: ***<0.001<**<0.01<*<0.05)

Table S11.C. SIMPER analysis results of conventional vineyard - natural area contrast for the *Lasius paralienus* CHC profile management variable (ordered by decreasing average contribution of CHC peak to the average dissimilarity between observations from the two groups).

Peak	Average	Standard deviation	ratio	Average		cumsum	p-value
				C_M	N_M		
20	0.00874745	0.00674151	1.29755083	2.84738794	3.0904017	0.03237018	0.35923
35	0.00843068	0.00665016	1.26774036	2.92644305	3.14884496	0.06356815	0.4714
37	0.00806163	0.00593379	1.35859719	2.00320788	2.24958819	0.09340044	0.40761
33	0.00790621	0.00533324	1.48243933	2.57154836	3.09203049	0.12265759	0.37113
18	0.00775321	0.00578917	1.3392619	2.22963451	2.65736994	0.15134857	0.39811
19	0.00771209	0.00631427	1.22137416	1.56731111	1.70465376	0.17988737	0.28201
42	0.0077012	0.00697195	1.10459802	2.48049226	2.94612665	0.20838588	0.47761
32	0.0075781	0.00648718	1.16816654	2.6529814	2.78783656	0.23642888	0.6347
38	0.00740895	0.00561271	1.32003161	1.90558443	2.27423347	0.26384592	0.50267
31	0.00732951	0.00749612	0.97777478	2.94424	3.49334275	0.290969	0.42689
44	0.00730903	0.00468486	1.56013971	1.15281126	1.62010591	0.31801629	0.33308
34	0.00719897	0.00567196	1.26921947	1.88282618	2.09219356	0.34465628	0.4327
2	0.00714973	0.00537386	1.33046496	2.15019135	1.55088826	0.37111407	0.54676
24	0.00714124	0.00551817	1.29413124	1.62699754	2.05041065	0.39754043	0.36241
22	0.00710749	0.00503278	1.41223859	1.38220621	1.67256205	0.42384191	0.29441
28	0.00707066	0.00502913	1.40593983	1.18120343	1.46753847	0.4500071	0.15681

29	0.00685664	0.0059554	1.15133058	2.61445529	2.95428467	0.47538029	0.49246
17	0.00682787	0.00552957	1.2347918	1.64121346	1.65066705	0.50064703	0.39122
41	0.00644813	0.00501528	1.28569827	1.93093442	2.14878074	0.52450854	0.66414
43	0.00642899	0.00486566	1.32129762	1.23415195	1.45582806	0.54829922	0.69426
14	0.0064253	0.00542329	1.18476172	1.77561172	2.1060541	0.57207624	0.25507
36	0.00625231	0.00432718	1.44489242	1.28048308	1.55085973	0.5952131	0.38208
25	0.0062394	0.00429188	1.45376651	1.2392122	1.37171743	0.61830217	0.33346
12	0.00611266	0.00513445	1.19052067	2.30397143	1.77999124	0.64092227	0.1646
21	0.00603829	0.00423277	1.42655754	1.33713591	1.54532747	0.66326713	0.33596
26	0.00573191	0.00390276	1.4686809	0.98652938	1.17633759	0.68447825	0.25278
16	0.00569617	0.00556407	1.02374117	1.01521779	1.43093644	0.70555708	0.29504
30	0.00565242	0.00418779	1.34973692	1.83217878	1.93309532	0.72647401	0.50597
13	0.00564628	0.00410052	1.37696627	0.69798633	0.98178354	0.74736823	**0.0442***
45	0.00544423	0.00379471	1.43468713	0.97110207	1.1468143	0.76751475	0.66739
27	0.00530528	0.00339841	1.56110541	1.30188995	1.2299761	0.78714708	0.26052
15	0.00529006	0.00433555	1.22015847	1.24725661	1.49327501	0.80672311	0.88024
39	0.00498323	0.00376717	1.32280596	0.65209041	0.6587579	0.82516369	0.3044
49	0.00493975	0.00416879	1.18493627	1.05764208	1.36714621	0.84344339	0.68662

7	0.00457477	0.00334398	1.36806053	0.75116682	0.88838826	0.86037247	0.14766
40	0.00403876	0.00282478	1.42976143	0.57446472	0.60784478	0.87531801	0.32844
23	0.00393656	0.00298072	1.32067065	0.64815939	0.64900743	0.88988535	0.25181
48	0.00370779	0.00265768	1.39512215	0.61346474	0.79434265	0.90360615	0.72475
8	0.00357725	0.0024897	1.43682079	0.5638115	0.68567954	0.91684387	0.31065
47	0.00330277	0.0027055	1.22076197	0.53021789	0.72900315	0.92906586	0.44896
11	0.00314427	0.00247915	1.26828816	0.34427822	0.55559365	0.94070134	0.25137
6	0.00278144	0.00222542	1.24984847	0.7979346	0.59179162	0.95099414	0.86545
4	0.00270531	0.00257645	1.05001417	0.31998293	0.45604464	0.9610052	0.28318
9	0.0026608	0.00174389	1.52578307	0.3076339	0.52296087	0.97085158	0.51115
10	0.0022504	0.00165071	1.36328888	0.32992903	0.26819019	0.97917925	0.5156
46	0.0021799	0.00208148	1.04728707	0.35194845	0.2284312	0.98724606	0.47592
5	0.00139027	0.00127302	1.09210461	0.15876532	0.17414684	0.99239081	0.35726
3	0.00112164	0.00089818	1.24879336	0.18199415	0.13436817	0.99654147	0.51018
1	0.0009346	0.00072405	1.29080648	0.19678679	0.13957687	1	0.99592

*/**/***: Asterisks denote significant p-values (p: ***<0.001<**<0.01<*<0.05)

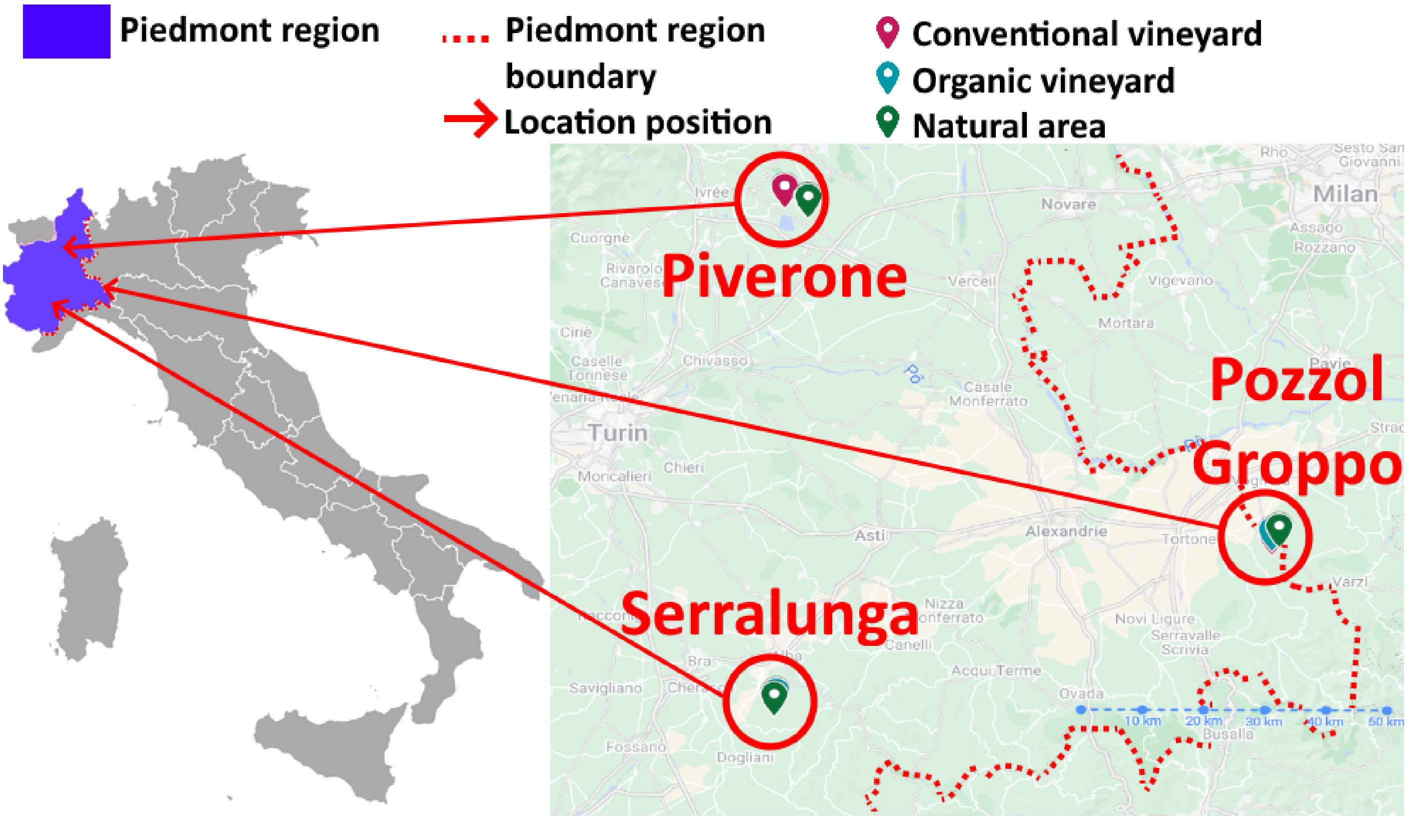

Figure S1. Italian map with locations and managements localizations inside the Piedmont region

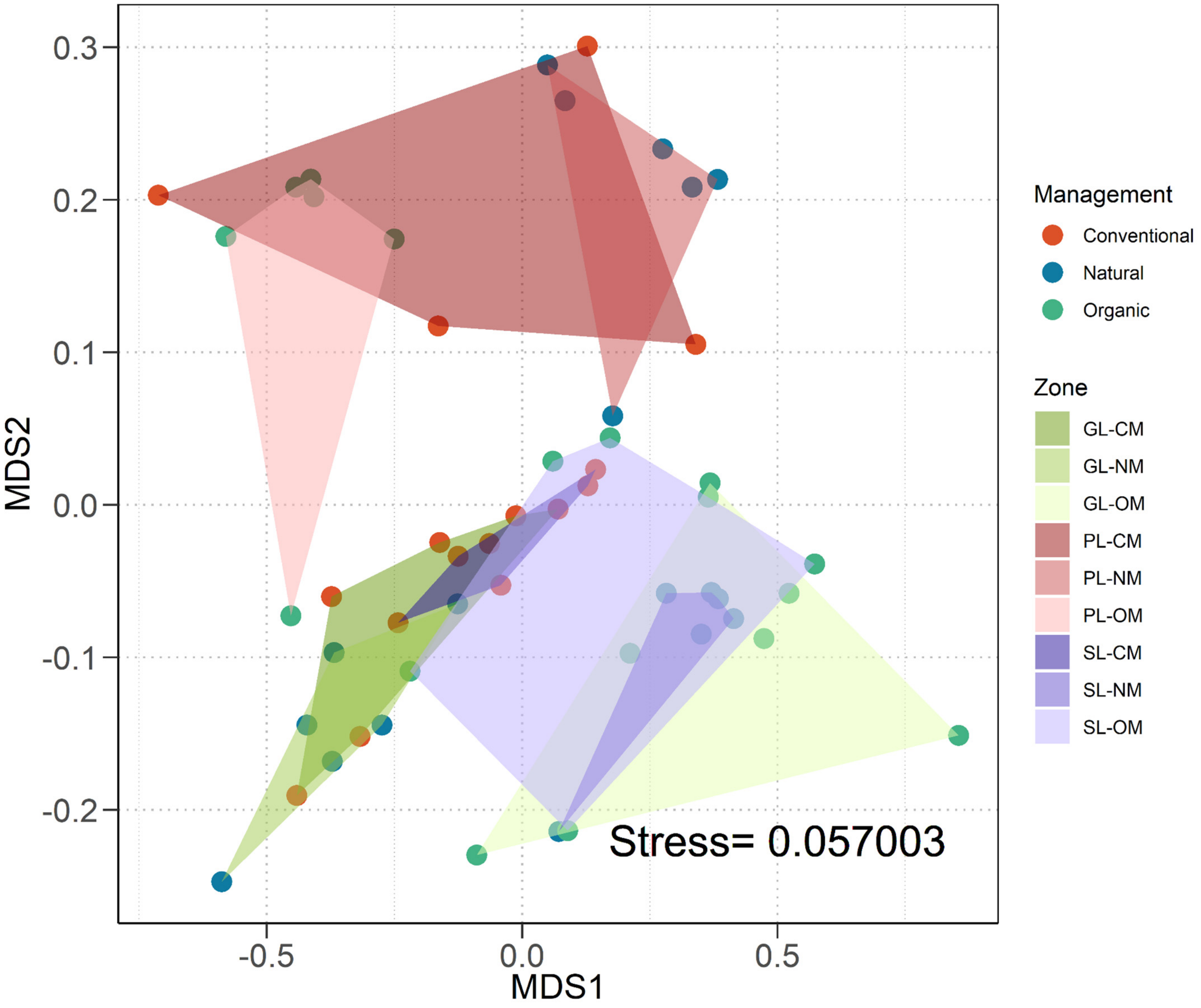

Figure S2. Non-metric multidimensional scaling plots (k=2) of the soil properties depending on the management and location combination areas (based on the first and second MDS vectors)

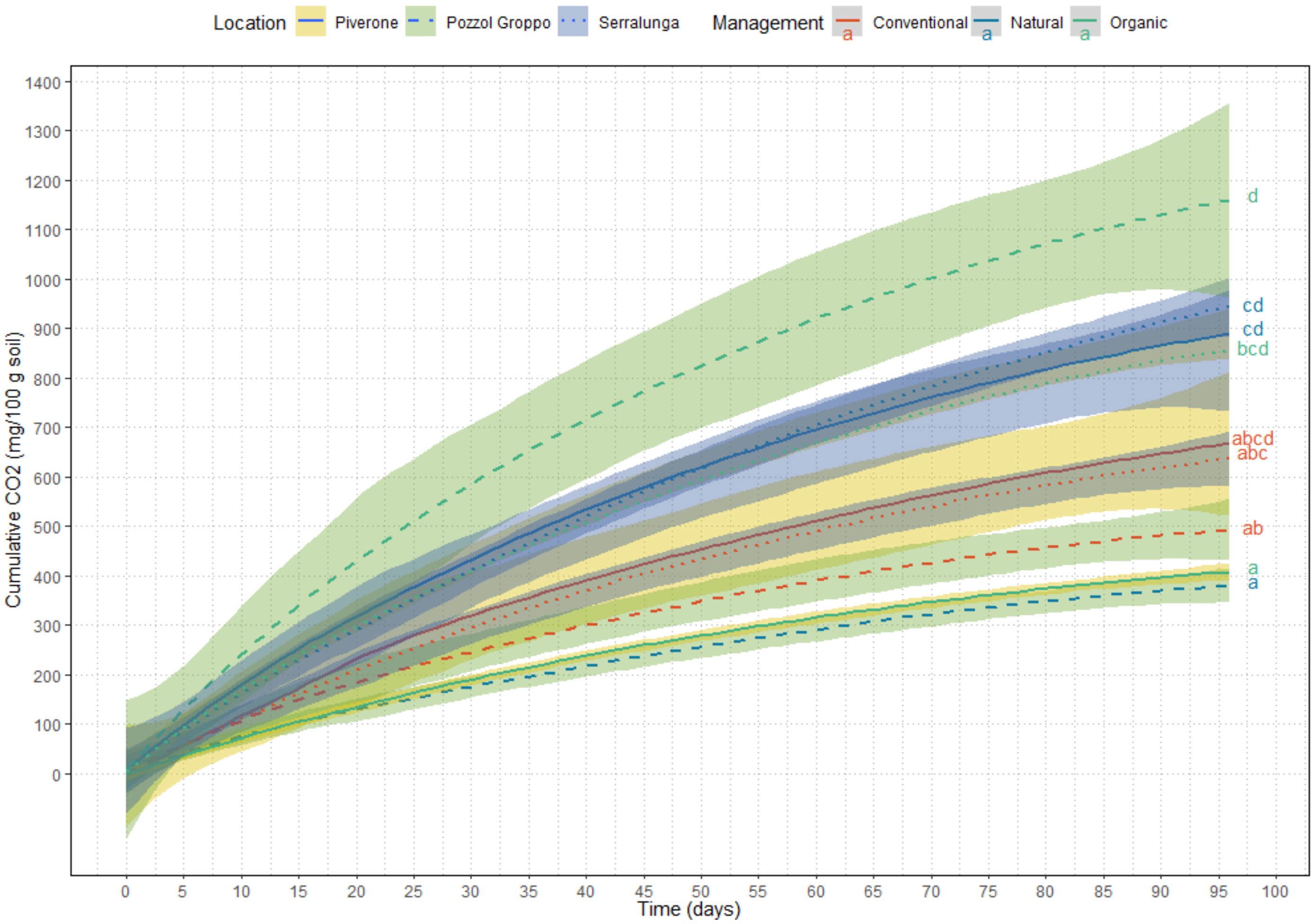

Figure S3. Soil biological activity or bSR, measured as CO_2 efflux (mg of CO_2/100 g of dry soil) in one incubation experiment (spring season) from the location and management combinations. Curves with different letters are representing statistically different expired CO_2 accumulated values of the entire incubation period, according to GLM pairwise comparisons.
